# Computational Modeling of Oxidative Stress in Fatty Livers Elucidates the Underlying Mechanism of the Increased Susceptibility to Ischemia/Reperfusion Injury

**DOI:** 10.1016/j.csbj.2018.10.013

**Published:** 2018-11-01

**Authors:** Jana Schleicher, Uta Dahmen

**Affiliations:** a Experimental Transplantation Surgery, Department of General, Visceral and Vascular Surgery, University Hospital Jena, Jena, Germany; b Department of Bioinformatics, Friedrich Schiller University Jena, Jena, Germany

**Keywords:** Steatosis, Hepatic fatty acid metabolism, Oxidative stress, Reactive oxygen species, Lipid peroxidation, ALOX12, Arachidonate 12-lipoxygenase, AOD, Antioxidative defense, cAMP, Cyclic adenosine monophosphate, CAT, Catalase, DNL, de novo lipogenesis, FA, Fatty acid, GPx, Glutathione peroxidase, GSH, Reduced glutathione, GSSG, Oxidized glutathione, HFD, High-fat diet, HIF, Hypoxia-inducible factor, H_2_O_2_, Hydrogen peroxide, 4HNE, 4-Hydroxynonenal, IL, Interleukin, IR, Ischemia/reperfusion, IRI, Ischemia/reperfusion injury, LPO, Lipid peroxidation, MDA, Malondialdehyde, NFκB, Nuclear factor kappa B, OH^⁎^, Hydroxyl radical, O_2_, Oxygen, O_2_^–^, Superoxide anion, 8-OHdG, 8-Hydroxydeoxyguanosine, ROS, Reactive oxygen species, TBARS, Thiobarbituric acid reactive substances, TG, Triglyceride, TNF, Tumor necrosis factor, UCP2, Uncoupling protein-2

## Abstract

**Question:**

Donor liver organs with moderate to high fat content (i.e. steatosis) suffer from an enhanced susceptibility to ischemia/reperfusion injury (IRI) during liver transplantation. Responsible for the cellular injury is an increased level of oxidative stress, however the underlying mechanistic network is still not fully understood.

**Method:**

We developed a phenomenological mathematical model of key processes of hepatic lipid metabolism linked to pathways of oxidative stress. The model allows the simulation of hypoxia (i.e. ischemia-like conditions) and reoxygenation (i.e. reperfusion-like conditions) for various degrees of steatosis and predicts the level of hepatic lipid peroxidation (LPO) as a marker of cell damage caused by oxidative stress.

**Results & Conclusions:**

Our modeling results show that the underlying feedback loop between the formation of reactive oxygen species (ROS) and LPO leads to bistable systems behavior. Here, the first stable state corresponds to a low basal level of ROS production. The system is directed to this state for healthy, non-steatotic livers. The second stable state corresponds to a high level of oxidative stress with an enhanced formation of ROS and LPO. This state is reached, if steatotic livers with a high fat content undergo a hypoxic phase. Theoretically, our proposed mechanistic network would support the prediction of the maximal tolerable ischemia time for steatotic livers: Exceeding this limit during the transplantation process would lead to severe IRI and a considerable increased risk for liver failure.

## Graphical Abstract

**Figure:**
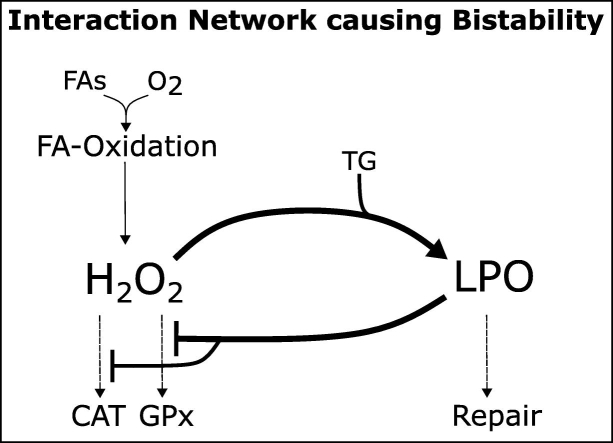

## 1 Introduction

The shortage of donor organs for liver transplantation called for the extension of donor organ criteria, so that suboptimal grafts, such as fatty livers, are more and more used for liver transplantation [1]. Moreover, the increasing prevalence of fatty livers in western populations leads to higher numbers of patients with fatty livers subjected to major liver surgery [2]. Fatty livers are characterized by an aberrant fat accumulation within the cytosol of hepatocytes (termed steatosis). Transplanting such fat-loaded livers is accompanied by a higher incidence of postoperative complications and transplant rejections leading to higher morbidity and patient mortality [3,4].

**Fig. 1:**
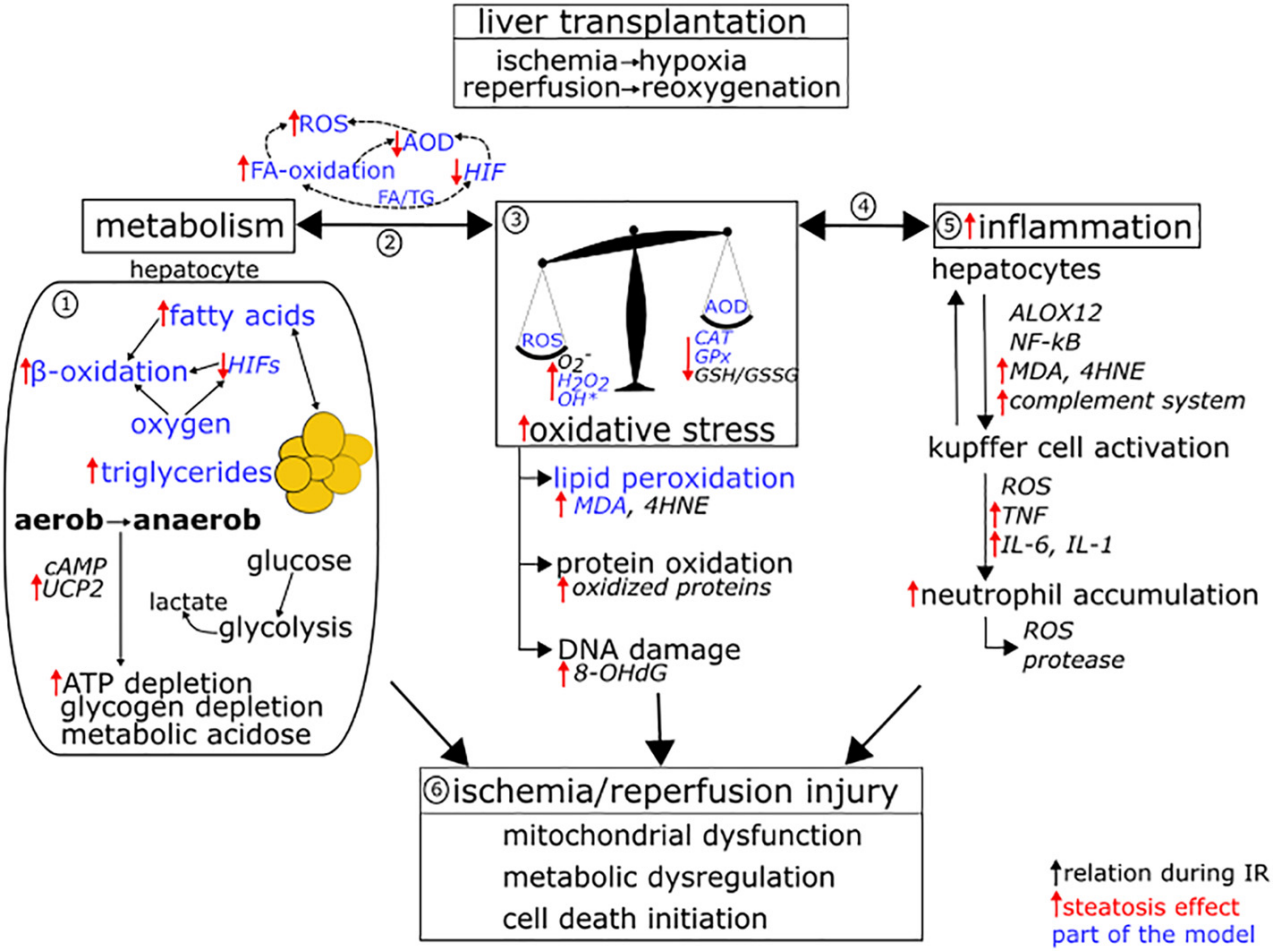
Overview of selected relevant factors and processes influencing the severity of ischemia/reperfusion injury (IRI) with focus on hepatic lipid and glucose metabolism, reactive oxygen species (ROS) formation, the antioxidative defense system (AOD) and inflammation. In sum, these processes mediate cellular damage during IR conditions, as encountered in donor organs during liver transplantation. Black arrows indicate relations between factors and processes crucial for IRI. Red arrows indicate the effect of a high grade of steatosis (i.e. excess of triglyceride, TG, accumulation), which may contribute to the increased susceptibility of steatotic livers for IRI. The mathematical model presented in this paper includes hepatic lipid metabolism and its relations to oxidative stress (blue marking). ① During ischemia, the deficiency in oxygen and nutrient supply to hepatocytes impairs oxidative degradation of glucose and fatty acids (FAs), which decreases ATP synthesis. The switch to anaerobic metabolism further intensifies ATP depletion and increases lactate formation resulting in metabolic acidosis [26]. Here, cyclic adenosine monophosphate (cAMP), which is a key second messenger controlling cellular metabolism [27], is affected during ischemia [28]. Moreover, the transcription factors hypoxia-inducible factors (HIFs) are master regulators during cellular hypoxia, whereas an excess of lipid accumulation interferes with HIF activation [22]. The uncoupling protein-2 (UCP2) induces mitochondrial proton leak and is upregulated in steatosis [29], which contributes to a greater ATP depletion in fatty livers. ②/③ The metabolic adaptations during ischemia entail an increased formation of ROS (esp. superoxide anion O_2_^−^, hydrogen peroxide H_2_O_2_, hydroxyl radical OH^⁎^). The increased ROS formation and the impairment of the AOD (catalase CAT, glutathione peroxidase GPx, ratio reduced/oxidized glutathione GSH/GSSG), particularly in steatotic livers, causes a high level of oxidative stress. Oxidative stress leads to lipid peroxidation (LPO; markers are malondialdehyde MDA and 4-hydroxynonenal 4HNE), protein oxidation (marker is the content of protein carbonyls) and DNA lesions (measured by 8-hydroxydeoxyguanosine 8-OHdG), all elevated in steatotic livers [25,[30], [31], [32]]. On the other side, the attack of ROS on mitochondrial structures leads to the dysfunction of metabolic processes. ④/⑤ ROS and the end products of LPO activates the inflammatory response mediated by e.g. arachidonate 12-lipoxygenase (ALOX12) [33], nuclear factor kappa B (NFκB) [34,35] and the complement system [36,37]. Ultimately this leads to an accumulation of neutrophils, which additionally release proteases and further ROS, thus contributing to cellular injury. ⑥ Altogether, metabolic adaptations, oxidative stress and inflammatory processes are intermingled, creating a vicious cycle leading to mitochondrial dysfunction, metabolic dysregulation and, finally, the initiation of cell death.

Liver grafts with moderate to high fat accumulation specifically show an increased susceptibility to intraoperative ischemia/reperfusion injury (IRI) [[4], [5], [6]]. IRI is triggered by a biphasic process, which activates a series of metabolic adjustments and signaling processes (Fig. 1). Ischemic injury originates from the interruption of blood flow, which is associated with an insufficient perfusion of hepatic tissue and, therefore, a reduced supply of cells with oxygen (O_2_; i.e. hypoxia). However, O_2_ is essential as electron acceptor in the respiratory chain. Consequently, hypoxic conditions caused by ischemia let the cells suffer from ATP depletion [7,8]. Eventually, the lack of O_2_ disrupts proper hepatic metabolic function and can trigger the initiation of cell death processes [[9], [10], [11]]. Additionally, restoration of blood flow (i.e. reoxygenation) after a period of ischemia places the cells at further risk for metabolic dysregulation and the induction of inflammatory processes [9,12]. Reperfusion aggravates the ischemic insult and may increase the risk for organ failure [9]. Due to the high incidence of steatotic donor organs, a substantial understanding of the key processes responsible for the lower tolerance of these livers to IRI is needed.

Despite intense research, we currently do not fully understand the reason why steatotic livers are more prone to IRI than normal livers. Certainly, the principal source can be attributed to the fat-induced metabolic impairments and the disturbed hepatic microcirculation caused by the swelling of the fat-laden hepatocytes [2,13]. However, on the cellular level one of the main forces driving IRI (Fig. 1) is an intense formation of reactive oxygen species (ROS; mainly superoxide anion O_2_^−^, hydrogen peroxide H_2_O_2_, hydroxyl radical OH^⁎^). An excess formation of ROS is a feature of hepatic oxidative stress, a condition of a serious redox imbalance [[14], [15], [16], [17]]. During transplantation, steatotic livers suffer particularly from excessive mitochondrial ROS production [18,19], an impaired induction of the antioxidative defense system (AOD) [20], mitochondrial uncoupling [21] and a disruption of the hepatic stress response to hypoxia mediated by the transcription factors HIFs (hypoxia-inducible factors) [22]. All elements together culminate in severe mitochondrial injury and high oxidative stress in steatotic livers [5,23], much higher compared to normal livers not overloaded with fat. Cellular damage arises from the high level of intracellular ROS, which causes modifications of DNA and oxidation of cell proteins, as well as initiation of the reaction chain for lipid peroxidation (LPO) [24]. Although it is well-known that the fat-induced pathological changes in microcirculation and metabolism get further aggravated by ischemia/reperfusion (IR) [25], the underlying mechanism of the excessive mitochondrial ROS formation in steatotic livers under these conditions is not yet clear.

Here, computational modeling unifying the current knowledge about relevant physiological processes of ROS production and detoxification linked to hepatic fat metabolism will promote our understanding of ischemic injury in steatotic livers. A mathematical model that allows for the *in silico* simulation of hypoxia and reoxygenation for various degrees of steatosis would be particularly helpful.

In this paper, we introduce a mathematical model that links key processes of hepatic lipid metabolism to the formation and detoxification of ROS. The model allows the simulation of hypoxia and reoxygenation conditions and predicts the level of hepatic LPO as a marker of damage caused by oxidative stress. We reveal that the increased susceptibility of steatotic livers to IR can be explained by a feedback loop between processes of H_2_O_2_ detoxification and LPO production. This interaction pattern can finally cause a bistable systems behavior in the level of oxidative stress. Here, the first state represents a low level of oxidative stress and occurs in normal, low fat-laden livers, whereas for steatotic livers the system drives to the second state with a high level of oxidative stress. This modeling result promotes our understanding of the increased vulnerability of steatotic livers to IRI. Theoretically, our proposed mechanism would support the prediction of a maximal tolerable ischemia duration for steatotic livers: Going over this threshold would increase drastically the risk for severe IRI and liver failure.

## 2 Methods

We developed an integrated, mathematical model of the key pathways of lipid metabolism coupled with ROS metabolism (Fig. 1) using the Software R [38]. Here, the focus was put on well-known interactions between hepatic fat content and oxidative processes. The metabolic processes were implemented as rate laws based on current literature knowledge (all model details are provided in the Appendix A). The model allows the simulation of liver metabolism under normoxia and hypoxia followed by reoxygenation. Thus, it can be applied to elucidate interactions between fat and ROS metabolism under ischemia-like conditions (i.e. cellular hypoxia). We did not specifically include processes leading to reperfusion injury, e.g. the activation of inflammatory processes, additional to the ischemic injury. The simulation of reoxygenation after a period of hypoxia is focused on how the ischemic injury and the level of oxidative stress is augmented under reoxygenation. Therefore, our model aims to elucidate basic mechanisms of ischemic injury and how the level of cell damage propagates during reoxygenation.

For model development, we applied a modular approach by starting with the implementation and calibration of a stand-alone submodel of hepatic lipid metabolism, which is capable to simulate hepatic triglyceride (TG) accumulation for different levels of plasma fatty acid (FA) supply (i.e. low to high-fat diet, HFD). In a second step, ROS metabolism and known interactions with FA metabolism and LPO were integrated. LPO leads to the production of toxic intermediates such as malondialdehyde (MDA) and 4-hydroxynonenal (4HNE) [39]. Therefore, in our model, the level of LPO is assessed by the hepatic concentration of MDA, which is typically used as an indicator of LPO damage in biological and medical sciences [40]. The hepatic concentration of MDA is typically determined by a TBARS assay (thiobarbituric acid reactive substances) [40].

The integrated model, finally providing a system of 5 ordinary differential equations, was calibrated and validated using current literature data. For simulation and model analysis we used the R packages ‘deSolve’ [41] and ‘FME’ [42]. The R code of the mathematical model is provided in Appendix D.

The robustness of our model output was tested by considering a 10% variation (i.e. 10% standard deviation) for each parameter. To show the effects of such variation in parameter values, we conducted 100 runs with different parameter values. Before a run, for each parameter a value was drawn randomly from a normal distribution with the value from the original model as mean and a 10% standard deviation. The emerging patterns under normoxic conditions were recorded. In addition, to specifically consider the robustness in MDA formation as output of our model, we looked at the pattern emerging under a higher parameter variation in this process. For this, we changed the parameter *k*_*MDA*_, involved in MDA formation, by 50% and investigated the systems behavior under normoxic conditions.

Our phenomenological model allows a closer look on the consequences of temporal hypoxia on fat metabolism, oxidative stress level and LPO production in the liver. Our intention was not to construct a comprehensive representation of each mechanistic detail of hepatic metabolism and, therefore, our model does not allow simulating a daily time course of lipid compounds. Rather, we put emphasize on the phenomenological simulation of hepatic lipid metabolism and oxidative stress regarding the amount of stored fat metabolites.

### 2.1 Lipid Submodel

A mathematical model, representing key processes of FA metabolism in the liver, was established based on previously published models [43,44] with some modifications. In our model, the following pathways are represented by rate laws (mass action kinetics and modified Michaelis-Menten kinetics): FA and O_2_ uptake from the blood into the liver cells, mitochondrial FA oxidation, a term representing other oxidative processes, TG synthesis and export. Details of the mathematical model and its equations, as well as parameter values, can be found in Appendix A and Table A1. Parameter calibration (see Appendix B) and validation of the metabolic model (see results Section“3.1 Model validation”) was conducted using experimental data from literature.

For simulation runs, we used a range from 0.1 mM to 1.4 mM of plasma FA concentration [[45], [46], [47], [48]] as model input. This range covers a normal diet up to a chronic HFD, respectively. In our model, the supply of FAs via blood determines the accumulation of TGs within the liver cells, thereby determining the severity of steatosis. Of note, we do not directly model plasma TG circulation. Simulation runs started at normoxic conditions and the model was executed until the state variables reached a stable steady state. Starting from this stable state, the model was executed under hypoxic conditions (i.e. the O_2_ concentration in blood was reduced to hypoxia-like conditions, see Appendix A2 for details) until, again, a stable steady state was reached. Reoxygenation was then simulated by setting the O_2_ supply back to the normoxic value.

### 2.2 ROS Submodel

The lipid submodel was extended by equations representing hepatic ROS formation and detoxification by antioxidative enzymes. We focused on H_2_O_2_ because it is more stable than the superoxide anion O_2_^−^. The toxicity of O_2_^–^ is principally based on the generation of further ROS (mainly OH^⁎^), which then attacks biomolecules [49]. Furthermore, H_2_O_2_ generation in hepatocytes seems to be mainly independent from O_2_^–^ production by the respiratory chain but depends in major parts on the activity of FA oxidation [50,51]. Thus, we decided to implement H_2_O_2_ and OH^⁎^ production. In our model, the production of H_2_O_2_ depends directly on intracellular O_2_ concentration and the rate of FA oxidation. ROS production under hypoxic conditions does not directly mirror mitochondrial respiratory chain activity [52], thus we focused our model on ROS production by mitochondrial β-oxidation of FAs.

The implementation of rate equations for the antioxidative enzymes catalase (CAT) and glutathione peroxidase (GPx) follows previously published models [53,54]. Here, the inhibition of CAT activity by a high concentration of its substrate H_2_O_2_ is accounted for by an inhibition term [53].

The concentration of H_2_O_2_ directly affects the production rate of OH^⁎^, which is the most important ROS regarding cellular damage due to its high reactivity [24]. This radical oxidizes intracellular lipids, thereby initiating LPO. The process proceeds as free radical chain reactions leading to the production of toxic intermediates such as MDA [39]. As mentioned above, the level of LPO is assessed by the concentration of MDA in our model. The level of oxidative stress can be assessed by the degree of H_2_O_2_ production.

To simulate ischemia-like conditions, we also need to account for hypoxia-induced (regulatory) effects in our metabolic model. The oxidation rate of FAs is influenced under hypoxic conditions by the expression of HIFs [55], which mediate metabolic adaptations during phases of O_2_ paucity [56]. A decreasing O_2_ concentration leads to a switch-like response of HIF activation with a plateau at very low O_2_ levels [57]. To account for the effect of hypoxia in our modeling framework, we adjusted the equation of mitochondrial FA oxidation by adding a sigmoidal term depending on intracellular O_2_ concentration. Further details of rate equations and parameter values are provided in the Appendices A2, B2 and Table A1.

## 3 Results

### 3.1 Model Validation

**Fig. 2:**
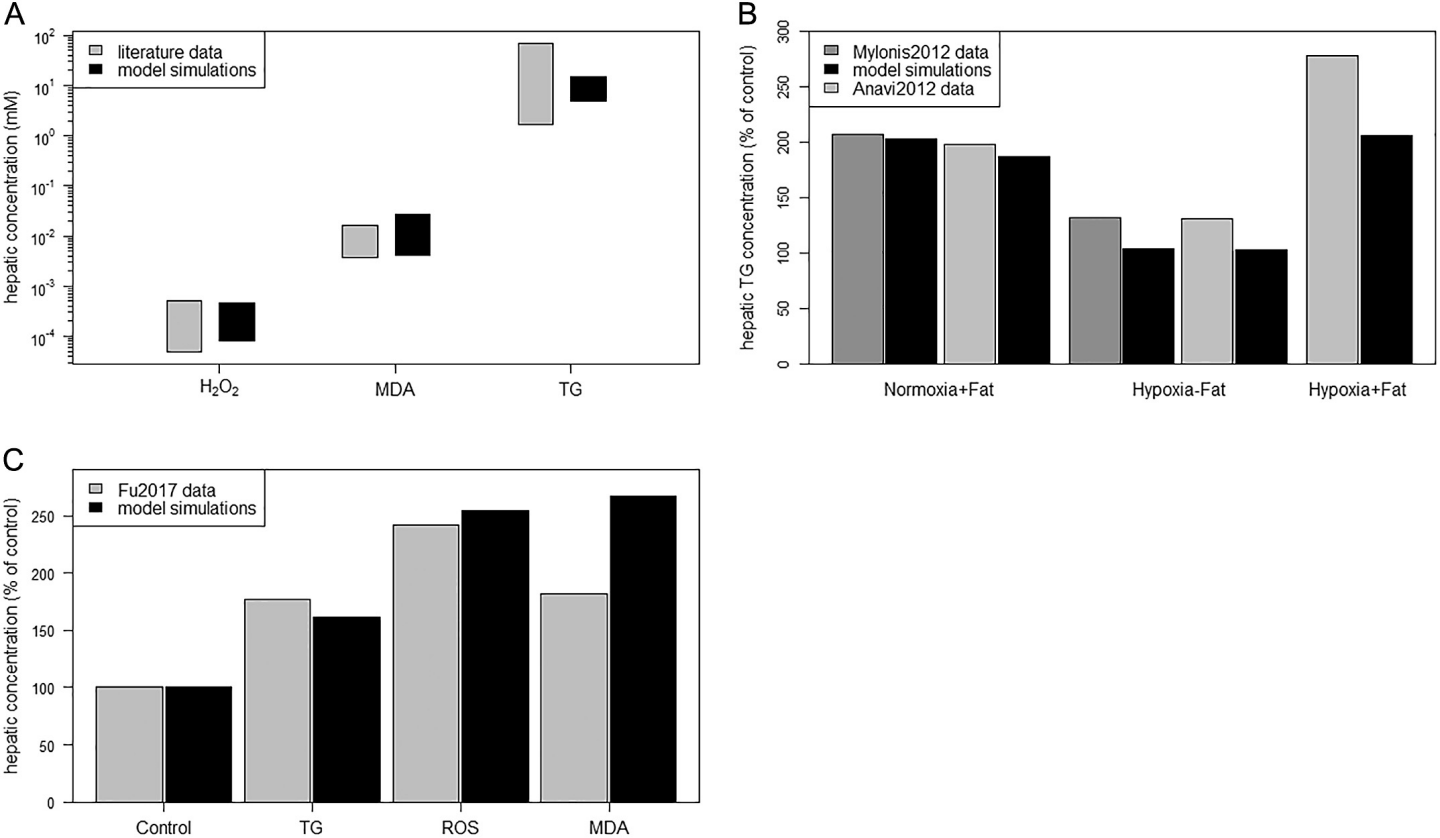
Model validation by comparison of simulation results to different data extracted from literature. (A) Comparison of reported hepatic concentrations to simulated (steady-state) concentrations. Simulations were conducted over the whole range of plasma fatty acid (FA) concentrations (0.1 mM–1.4 mM, [45,48]). Typical hepatic concentrations of malondialdehyde (MDA), a marker of lipid peroxidation (LPO) [[59], [60], [61]], and triglycerides (TGs) [62,63] for normal and steatotic livers were extracted from literature. Typical hepatic concentrations of hydrogen peroxide (H_2_O_2_) in normal livers were also found in literature [14,64], but no reports on concentrations in steatotic livers could be found. Therefore, we used values reported by Sies [65] (up to 5 × 10^−4^ mM) to be typical for livers under stress conditions, which is in good agreement to values reported for livers after ischemia/reperfusion (around 4 × 10^−4^ mM) [14]. (B) Comparison of model simulations to literature data of hepatic TG accumulation under normoxia and hypoxia. Data of TG content for human hepatoblastoma (Huh7) cells were adapted from Fig. 1C in Mylonis et al. [66] and for AML12 hepatocytes from Fig. 1A in Anavi et al. [22]. In the experimental study by Mylonis et al. [66], cells were incubated at normoxia (20% oxygen (O_2_)) and hypoxia (1% O_2_) in a low-fat medium. Additionally, 0.4 mM of oleic acid was added to the medium (+Fat condition). Accordingly, model simulations were conducted with 0.1 mM FA supply (*FA*_*blood*_) under normoxia and hypoxia conditions, respectively. Normoxia under low FA supply was used as control and set to 100%. We repeated the normoxia simulation scenario with 0.5 mM FA supply to compare our simulation results with the normoxia + fat data. In the experimental study by Anavi et al. [22], hepatocytes were also cultured under normoxic (21% O_2_) and hypoxic (1% O_2_) conditions for low and high fat supply. Accordingly, model simulations were conducted with 0.2 mM and 1.0 mM plasma FA concentrations under normoxic and hypoxic conditions, respectively. Normoxia under low FA supply was used as control and set to 100%. (C) Comparison of literature data and model simulations for the accumulation of TGs, reactive oxygen species (ROS) and MDA under a high-fat diet (HFD). Data were extracted from Fu et al. [67], who fed one group of mice with a standard chow as control and a second group of mice with a high-lard/high-cholesterol diet to induce a fatty liver in the animals. The authors measured hepatic TG and ROS content as well as the MDA level (see [67]). Model simulations were conducted with 0.7 mM plasma FA concentration, representing a HFD like in the study. For control simulations we used 0.2 mM plasma FA concentration.

We validated our phenomenological model by comparison of simulation results with a broad range of experimental data (Fig. 2A-C) and known patterns (Table 1) extracted from various literature sources. Numerical data only reported in figures and plots were extracted via WebPlotDigitizer [58]. The data for model validation is different from the data used for model calibration.

**Table 1:** Comparison of observed biological patterns to model output. Abbr.: FA – fatty acid, GPx – glutathione peroxidase, HFD – high-fat diet, H_2_O_2_ – hydrogen peroxide, IR – ischemia/reperfusion, LPO – lipid peroxidation, MDA – malondialdehyde, O_2_ – oxygen, TBARS – Thiobarbituric acid reactive substances, TG – triglyceride.

Validation issue	Model simulation	Experimental observation
Intracellular O_2_ concentration (normoxia)	Range of intracellular O_2_ concentrations during normoxic simulations is between 0.22 mM–0.27 mM^a^	Physiological O_2_ concentration is up to 250 μM (0.25 mM) [68]
Modifying FA uptake rate	Enhancing the rate of FA uptake (via increased value of parameter *k*_*FAup*_) raised hepatic TG content up to ~200%, TG synthesis and TG export up to ~120%	CD36 overexpression enhances the rate of FA uptake, which increases hepatic TG content to ~190%, TG synthesis to ~130% and TG export to ~120% (compared to control) [69]
Modifying mitochondrial FA oxidation	Decreasing the rate of FA oxidation (via decreasing the parameter value *k*_*oxid*_) causes an increased accumulation of TG	Inhibition of mitochondrial oxidation by tetracycline increases hepatic TG content [70]
Effect of a HFD	Strong increase of the intracellular FA concentration from low to high plasma FA supply (between ~ 300% to ~500%^a^); increase of H_2_O_2_ production from low to high plasma FA supply (between ~140% to ~170%^a^); increase of GPx activity (between ~120% to ~175%^a^) and MDA concentration (between ~170% to 300%^a^) from low to high plasma FA supply	~300% increase of intracellular FA concentration under a HFD in Wistar rats (compared to control diet) [71]; ~145% increase of H_2_O_2_ production under a HFD in Swiss mice (compared to control diet) [72]; GPx activity increases by ~195% and MDA content increases by ~150% under a HFD in rabbits (compared to control diet) [73]
Effect of hypoxia on H_2_O_2_ generation & hepatic FA & TG content	H_2_O_2_ production rate increases up to 200% of the normoxic value under hypoxia with moderate plasma FA supply; accumulation of intracellular FAs and TGs under hypoxia (see Fig. 6A, B)	H_2_O_2_ generation increases up to ~190% of control over 72 h of hypoxia [74]; Increased hepatic concentration of FAs and TGs under hypoxia [75]
LPO under HFD after IR	MDA concentration strongly increases after hypoxia & reoxygenation (2. stable state) under steatotic conditions	Level of TBARS strongly increases under a HFD compared to a standard diet after IR [76], similar observation directly for the MDA concentration [77]

a Results depend on the plasma FA concentration (parameter *[FA]*_*blood*_) chosen to represent the control and HFD, respectively.

### 3.2 Level of LPO and the Amount of FAs Trigger a Bistable Systems Behavior

Our novel constructed model, coupling lipid and ROS metabolism and using MDA content as model output, is based on mechanisms described in current literature (as delineated in Appendix A). It shows that the system can be directed in two distinct stable steady states, whereas the direction is determined by the initial level of LPO (represented by the concentration of MDA).

**Fig. 3:**
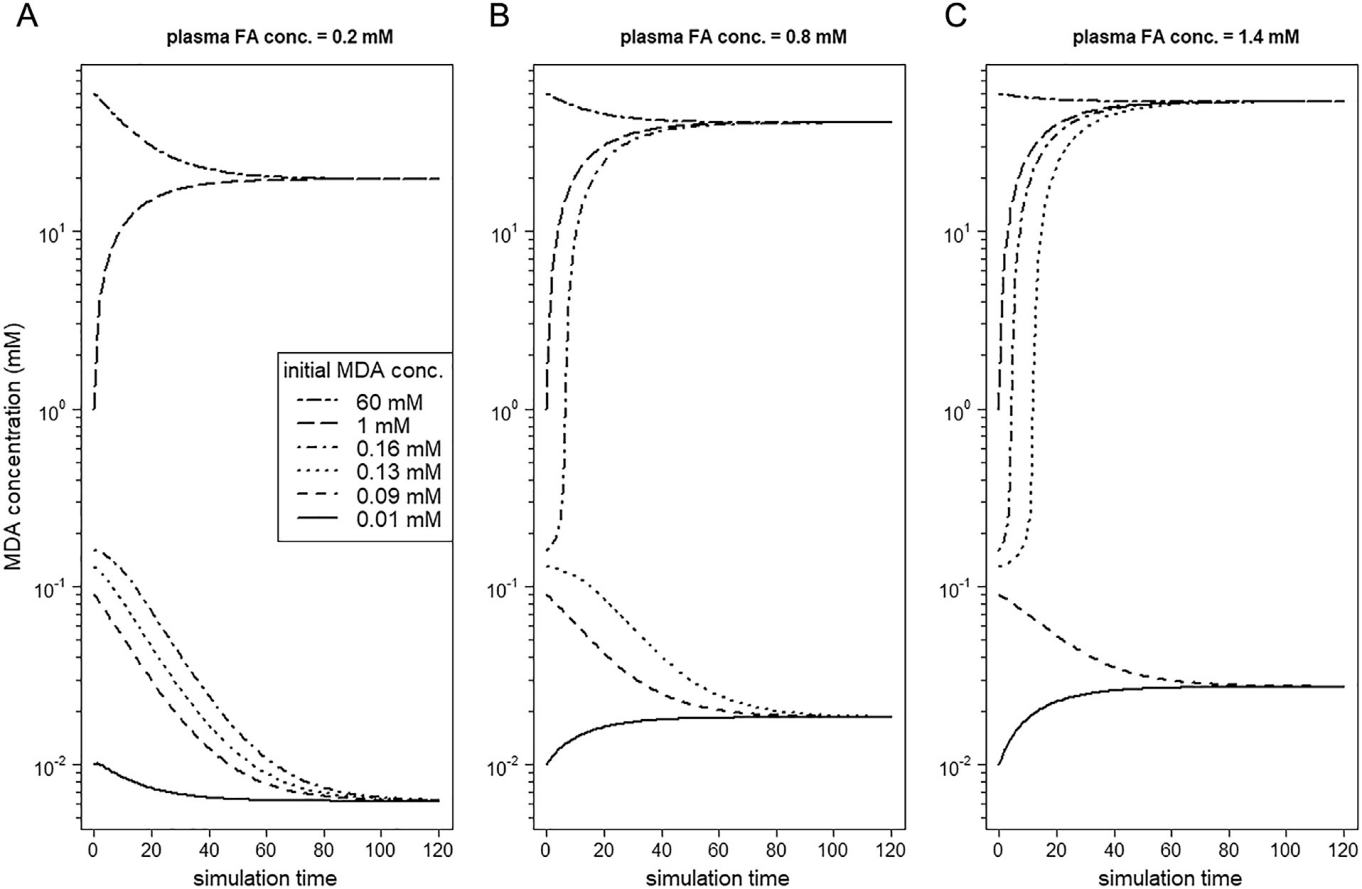
Initial malondialdehyde (MDA) concentration defines the steady state of the modeled system. Simulation runs of the modeled system under normoxic conditions starting from different initial MDA concentrations (which is a marker of lipid peroxidation) indicative of (A) low, (B) medium, and (C) very high fatty acid (FA) supply. The system shows bistability.

Starting from different initial concentrations of MDA (from zero to very high), we run our metabolic model under normoxia and with a fixed value of plasma FA concentration of 0.2 mM. Depending on the initial MDA concentration, the system reaches one of two stable steady states (Fig. 3A). If the initial level of MDA concentration is low, the ROS and MDA formation also stays in a low stable state. If the initial level of MDA concentration is high, the system is driven to a high level of oxidative stress (represented by H_2_O_2_ concentration, *data not shown*) and MDA. Thus, for the same parameter values the modeled system reaches a low and a high level of oxidative stress (low or high level of ROS and MDA formation, respectively).

Importantly, the concentration of supplied FAs via blood determines the threshold of MDA concentration, which must be exceeded to drive the system to the second stable state. Running the model for an increasing amount of plasma FA concentrations (Fig. 3A low – 0.2 mM, Fig. 3B medium – 0.8 mM, and Fig. 3C very high – 1.4 mM; note: y-axes are log transformed) revealed that the system under high FA supply (representing steatotic livers) reaches the second stable state for lower initial concentrations of MDA compared to low and moderate FA supply. This means that steatotic livers encounter a lower threshold of MDA to switch from a low to a high level of oxidative stress than normal livers. Moreover, in steatotic livers the MDA concentration in the state of low oxidative stress is higher than for normal livers (compare Fig. 3A and C), thus steatotic livers are undergoing more LPO.

### 3.3 Robustness of Bistability

**Fig. 4:**
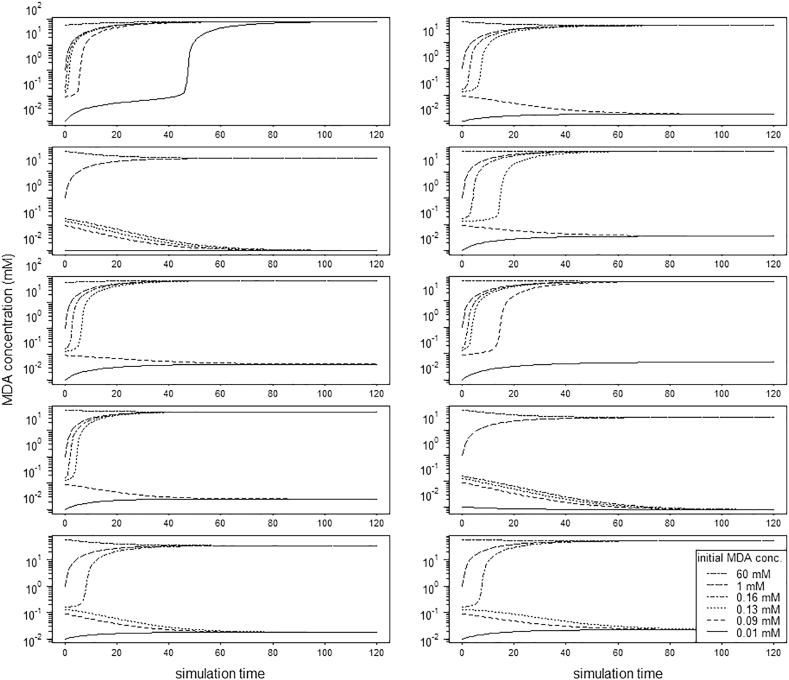
Robustness of the model: 10 out of 100 runs with randomly drawn parameter values from a normal distribution with 10% standard deviation. In 9 out of these 10 runs a bistable pattern emerged over the simulation time.

The output of a computational model may depend strongly on the chosen parameter values. Therefore, we considered the effect of a 10% standard deviation for each parameter to show the robustness of our model prediction. We conducted 100 runs with different parameter values each drawn from a normal distribution with the original value as mean and a 10% standard deviation. The model was run under normoxia for a range of MDA concentrations like the runs reported for the original parameter values in the results Section 3.2. The emerging system pattern was recorded (see an example of 10 runs in Fig. 4). The results of all runs are presented in Appendix C. In 95 out of 100 runs a bistable pattern emerged over the simulation time, showing the robustness of our model results regarding variation in parameter values.

**Fig. 5:**
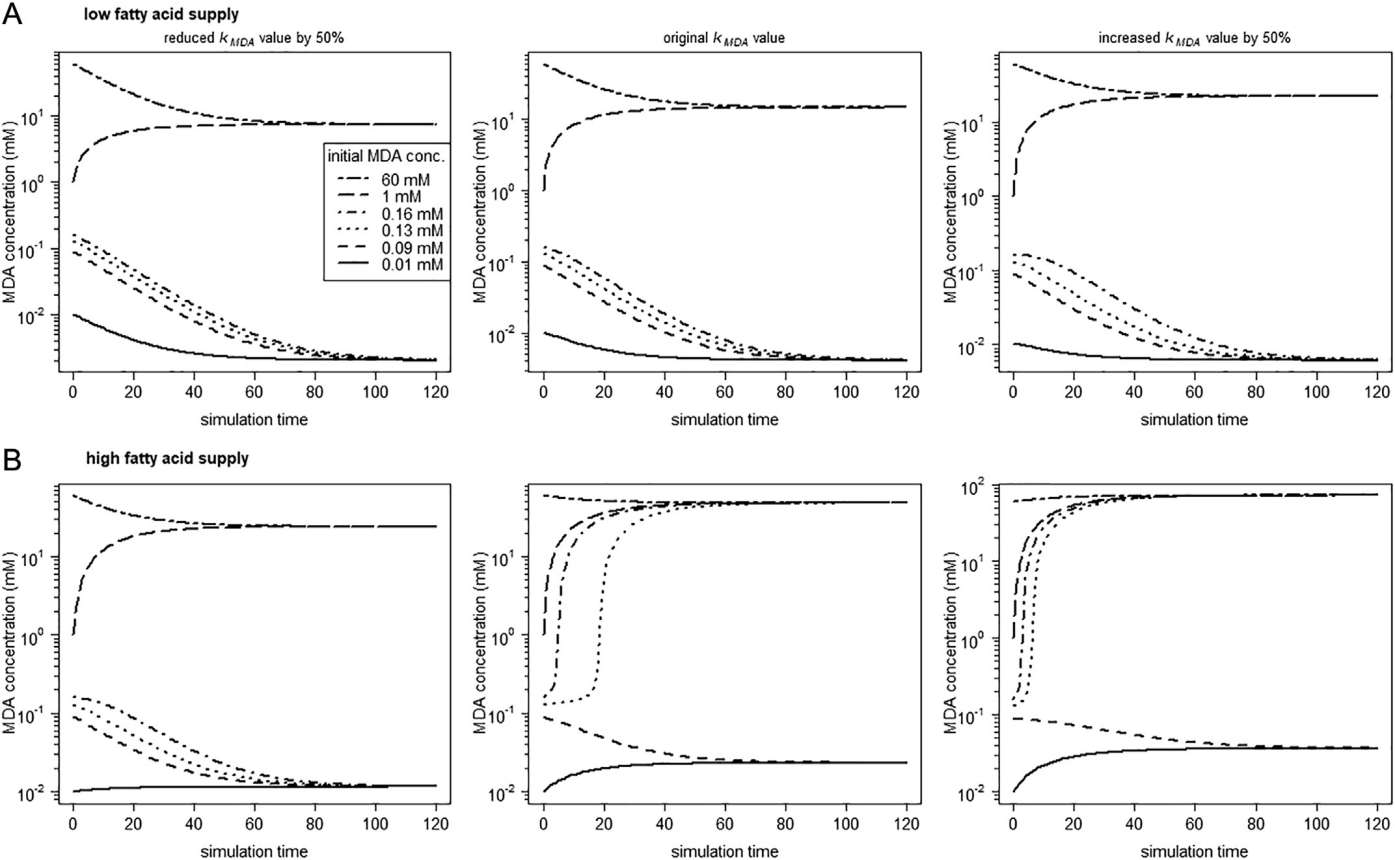
Under normoxia, MDA formation influences the threshold but not the systems behavior (i.e. bistability sustains). The parameter value of *k*_*MDA*_ (first order constant of the MDA production term) was reduced (to 0.15 mM^−1^) and increased (to 0.45 mM^−1^) by 50% of the original value (0.3 mM^−1^). Simulation runs were conducted under normoxia with (A) *FA*_*blood*_ = 0.1 mM and (B) *FA*_*blood*_ = 1.1 mM, respectively.

Furthermore, to evaluate how parameter variation in the MDA formation process may affect our model results, we changed the parameter value in MDA generation by 50%. We observed that increasing and decreasing of the *k*_*MDA*_ parameter value (Fig. 5) did not contradict our underlying model hypothesis of bistability. However, knowing the exact rate of MDA formation is essential to determine the threshold, at which the system shifts into the second stable state.

### 3.4 Hypoxia Enhances ROS and MDA Formation and Acts as Triggering Factor

Our novel model can be used to simulate the response of hepatic lipid and ROS metabolism under a lack of O_2_. So, we let the modeled system run under ischemia-like conditions (i.e. cellular hypoxia). Here, the observed bistable systems behavior matters and provides a basis for explaining the increased susceptibility of steatotic livers to hypoxia.

**Fig. 6:**
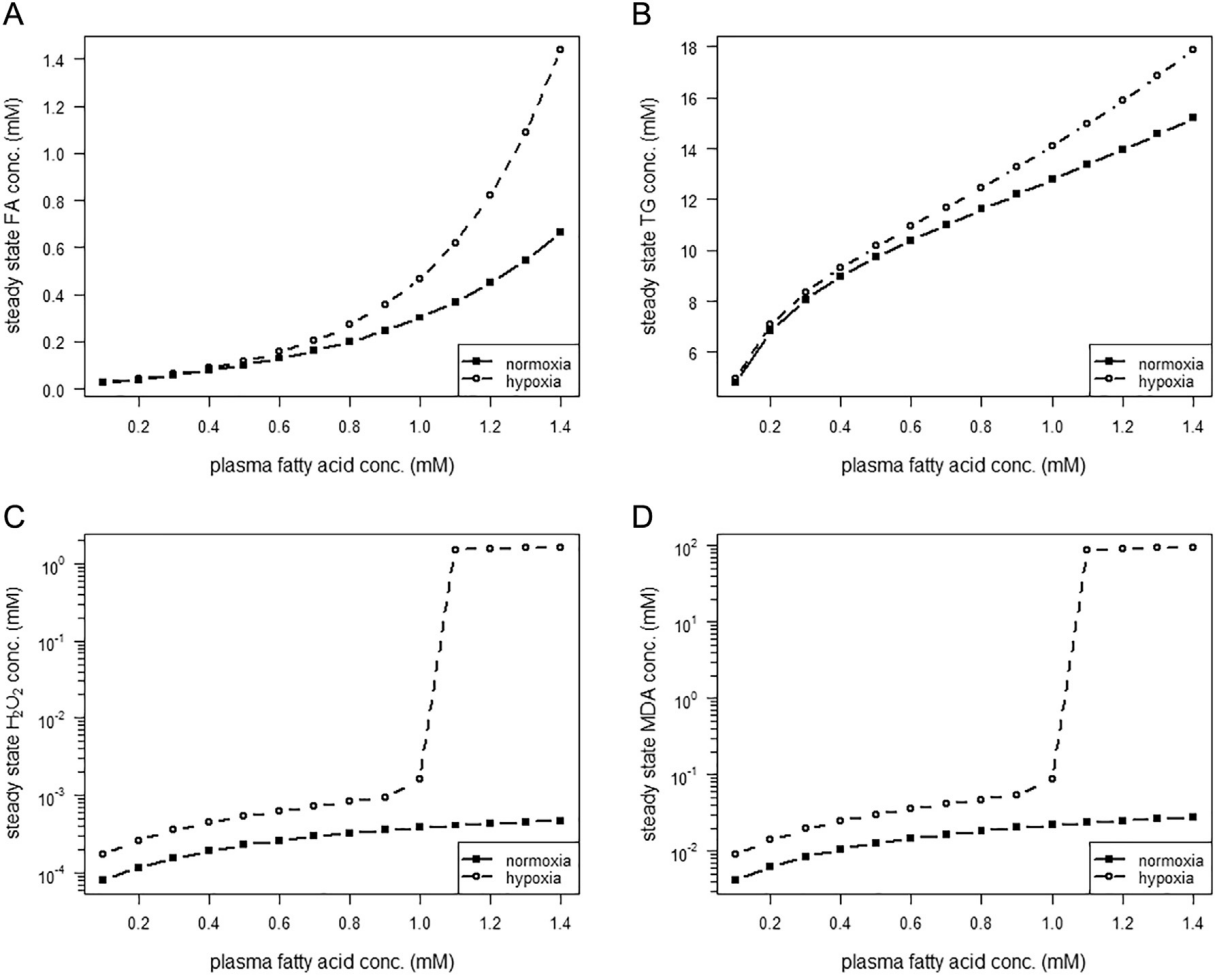
Steady state hepatic concentrations of state variables under normoxic and hypoxic conditions. Simulations under normoxia and hypoxia, respectively, were run to steady state of (A) intracellular fatty acid (FA) concentration, (B) intracellular triglyceride (TG) concentration, (C) intracellular hydrogen peroxide (H_2_O_2_) concentration (as marker of oxidative stress) and (D) intracellular malondialdehyde (MDA) concentration (as marker of lipid peroxidation).

Running the metabolic model under hypoxic conditions leads to a rise of the hepatic concentrations of FAs, TGs, H_2_O_2_ and MDA (Fig. 6) compared to the normoxic concentrations. Conditions with a high intracellular concentration of FAs and TGs (plasma FA supply >1.0 mM) cause a shift of the system to a high level of oxidative stress (Fig. 6C, note: y-axis is log transformed) and LPO (Fig. 6D, note: y-axis is log transformed). Finally, under steatotic conditions a lack of O_2_ supply leads to an increase of MDA formation above the threshold separating the first and second stable state. Thus, evoked by hypoxic conditions, the system is directed to a high state of oxidative stress.

### 3.5 Bistable Systems Behavior Explains Increased Susceptibility of Steatotic Livers to IRI

**Fig. 7:**
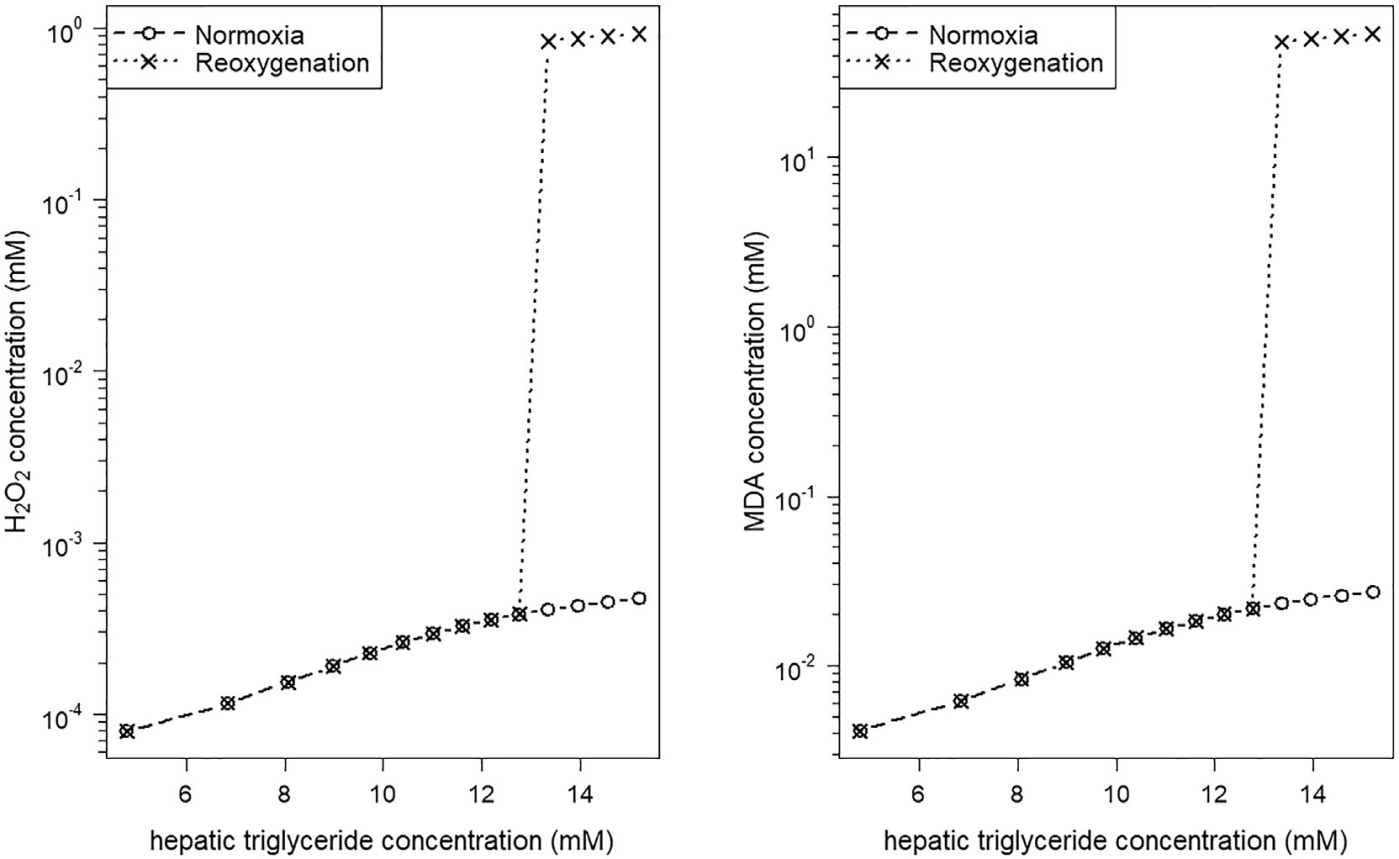
Hepatic concentration of hydrogen peroxide (H_2_O_2_) and malondialdehyde (MDA) increases in respect to the hepatic concentration of stored triglycerides (TGs). The steady state concentrations of H_2_O_2_ and MDA after simulation of transient hypoxia followed by reoxygenation are similar to the steady state concentrations under normoxic conditions for low to moderate hepatic TG concentrations. Livers with higher TG accumulation showed a sharp and stable increase of H_2_O_2_ and MDA concentrations after reoxygenation.

To evaluate how the system acts under reoxygenation after transient hypoxia, we rerun our model with normal O_2_ supply values starting from the steady state concentrations reached under hypoxia. After reoxygenation, the steady state hepatic concentrations of H_2_O_2_ and MDA reached values similar to normoxic conditions for low to moderate concentrations of stored TGs (Fig. 7). Note that the plasma FA concentration determines the level of stored TGs. Simulations in case of high TG concentration result in a great increase in the concentrations of H_2_O_2_ and MDA. This high level exceeds clearly the concentrations reached under normoxic conditions (Fig. 7). In contrast to these high H_2_O_2_ and MDA concentrations, the concentrations of the other model state variables (FAs, O_2_, TGs) are similar to the level under normoxic conditions (*data not shown*).

### 3.6 Duration of Hypoxia Influences the Level of Oxidative Stress

**Fig. 8:**
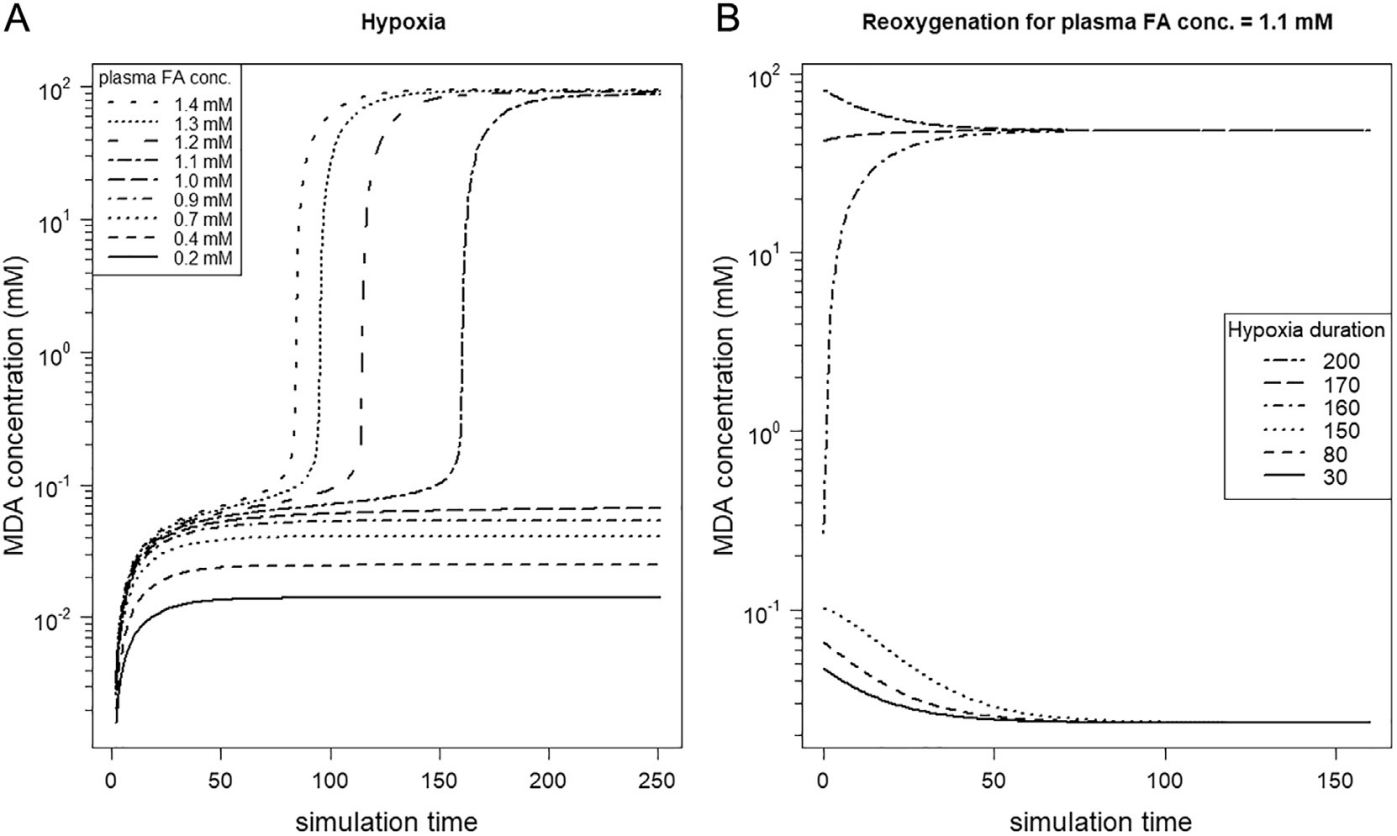
Duration of hypoxia determines state of oxidative stress depending on plasma fatty acid (FA) supply. (A) The concentration of FAs supplied via blood (parameter *FA*_*blood*_) influences the threshold at which the system jumps in the state of high oxidative stress. (B) Exemplified by a plasma FA concentration of 1.1 mM, the duration of the hypoxia phase determines, which state the system reaches after reoxygenation, a low or high oxidative stress state, respectively.

How does the duration of the hypoxic period influence the concentration of MDA? We started simulation runs over the whole range of plasma FA concentrations under hypoxic conditions. A low to moderate supply of FAs exhibited that the system stays in a low level of MDA, independent of the length of the hypoxic period (Fig. 8A for *FA*_*blood*_ = 0.2 mM–1.0 mM). However, for steatotic conditions (plasma FA concentration > 1.0 mM), the model outcome (i.e. MDA concentration) depends on the duration of hypoxia. A high supply of plasma FAs and, therefore, a high hepatic TG concentration, is associated with a MDA concentration exceeding the threshold. Thus, the system is driven to the second stable state. The higher the hepatic FA and TG concentrations, the earlier during hypoxia the threshold of MDA is reached, which directs the system to the high state of oxidative stress and LPO.

Exemplified by the simulation run with a plasma FA concentration of 1.1 mM (Fig. 8B), a short duration of hypoxia can be tolerated even by steatotic livers, but longer hypoxic periods force the system into the second stable state of high LPO. This pattern would allow the prediction of a certain cut-off for the maximal tolerable hypoxia duration depending on the observed hepatic FA and TG concentrations.

## 4 Discussion

Liver donor organs with a high fat content show increased susceptibility to IRI during transplantation. Up to now, there is no consensus on the risk of using steatotic liver grafts for transplantation and the question is still under debate how much fat accumulation is tolerable. The reasons for this are controversial results in studies reporting surgery outcome for transplantations of steatotic livers [79] and difficulties in the qualitative assessment of the amount and type of lipids in liver grafts [4]. The metabolic and signaling changes triggered by ischemia and aggravated during reperfusion are complex and strongly intermingled with the content of fat in the liver. It is known that steatotic livers exhibit an enhanced ROS formation overwhelming the AOD [78]. However, the interaction network between hepatic ROS formation, FA metabolism and LPO has to be elucidated. Thus, an understanding of the pathophysiological mechanisms in steatosis as well as the adaptations occurring under IR conditions is necessary to evaluate the risk of transplanting liver grafts with moderate to high steatosis grade.

We developed a mathematical model of hepatic lipid metabolism coupled to ROS metabolism. Model results show clearly a bistable systems behavior emerging from the underlying interaction network, driving the system into a low or high state of oxidative stress and LPO. Here, generally, keeping a low level of ROS and LPO is beneficially to cells because both act as signaling messengers [80]. On the other side, a high state of oxidative stress is the cornerstone of pathological conditions such as IRI [10] and nonalcoholic steatohepatitis [81].

The term bistability refers to a dynamic system that can stay stable in two distinct states. The switch from one stable state to the other stable state is triggered by (often external) stimuli, which do not need to be persistent. Bistability forms the basis for numerous phenomena in biological systems [82], among others in cell signaling [83,84], gene regulation [85,86], cell differentiation [87], regulation of apoptosis [88] and even in population dynamics [89]. A detailed mathematical model of the activity of the respiratory chain in mitochondria already uncovered a switch-like behavior from low to high ROS formation by the respiratory complex III [90]. In this model, the bistability in ROS formation is triggered by a lack of O_2_ (= anoxia), inducing a highly productive state of mitochondrial ROS formation. Consistent with our model results, the system stays in this high ROS formation state after reoxygenation. Generally, revealing a bistable pattern in a biological system provides a detailed view of how the system is regulated and what are the key components and their interrelations.

**Fig. 9:**
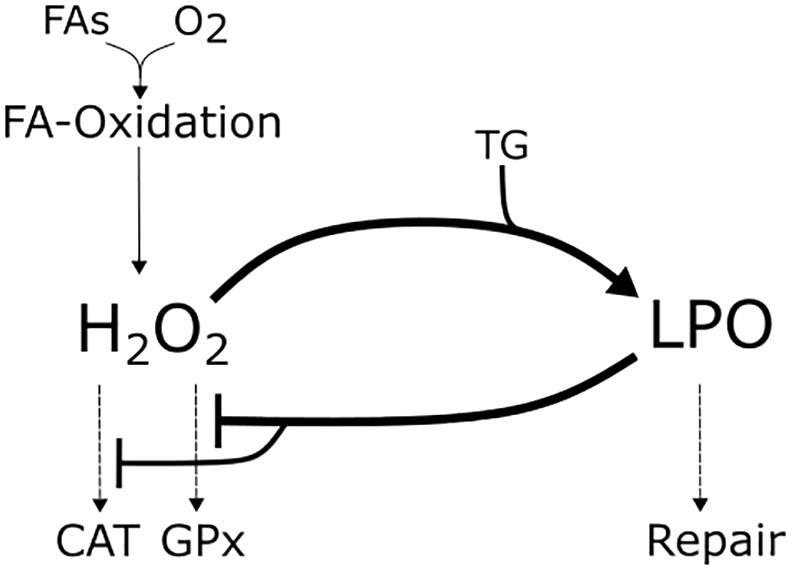
Overview of the interaction between hepatic lipid metabolism, hydrogen peroxide (H_2_O_2_) and lipid peroxidation (LPO) processes in hepatocytes. Mitochondrial oxidation, here represented by fatty acid (FA) oxidation under oxygen (O_2_) consumption, is associated with the formation of reactive oxygen species (here exemplified by H_2_O_2_). H_2_O_2_ is detoxified by the activity of catalase (CAT) and glutathione peroxidase (GPx). Importantly, H_2_O_2_ is converted to the highly reactive hydroxyl radical, which causes LPO of intracellular triglycerides (TG). The intracellular damage induced by LPO can be repaired to a certain extent by the hepatocytes or prevented by terminating the radical chain reactions. LPO inhibits the activity of the antioxidative enzymes CAT and GPx, therefore impairing H_2_O_2_ detoxification.

A biochemical system needs at least 3 structural elements to generate a bistable response [91]: (1) positive feedback, (2) a reaction to prevent explosion, and (3) a reaction to filter out small stimuli. All three elements can be found in the interaction network of hepatic ROS metabolism and LPO (Fig. 9), cumulatively determining the level of LPO. First, a positive feedback loop emerges in the formation of LPO determined by H_2_O_2_ (by the formation of OH^⁎^, see Appendix A2, Eq. 13) because LPO reduces the capacity of the AOD, which is responsible for H_2_O_2_ detoxification. The degradation of the AOD during IR [14] is well-grounded by the cytotoxicity of LPO and its end products. This includes the induced disruption of subcellular membrane structures [92] accompanied by alterations of membrane permeability, reduction of the glutathione level and enzymatic dysfunction [93]. This dysfunction is caused by the reaction of LPO end products with amino acids and proteins (causing alteration of enzyme structure and function, [94]) and DNA (modifying gene expression, [95]). Further support can be found by the lowered activity of the antioxidants observed in patients undergoing liver transplantation [96]. Second, the explosion of LPO is prevented by the termination of chain reactions and by cellular repair or protection mechanisms (i.e. antioxidants such as vitamin E, [97]). In the model, this was implemented by a repair equation representing the enzymatic metabolization of MDA [97]. Third and finally, small stimuli are filtered out by the activity of the AOD (here, CAT and GPx activity). GPx detoxifies H_2_O_2_ at relatively low concentrations, whereas CAT is active when H_2_O_2_ starts to accumulate [98,99]. Thus, if the rate of FA oxidation and therefore ROS formation gets slightly enhanced, e.g. after meals, the activity of CAT and GPx prevents an increase of H_2_O_2_ formation. Together, these 3 elements generate a bistable systems behavior in the level of oxidative stress and LPO.

The proposed bistable behavior provides a theoretical explanation for the increased susceptibility of steatotic livers to IRI. In our model, transient hypoxia enhances the formation of H_2_O_2_ and, thus, also the formation of MDA. If the MDA level exceeds its threshold, the system switches from the low to the high state of oxidative stress. For simulation runs with high concentrations of plasma FAs the threshold was reached already during a short period of hypoxia and forced the system into the stable state of high oxidative stress. The system stayed in this stable state also after reoxygenation and did not return to the low state of oxidative stress. This model behavior is in accordance to experimental studies revealing that the cell damage in IR experiments correlates with the duration of the ischemic period (see e.g. [100]). In case of a short ischemic period, the liver suffers only from cell injury that is reversible, thus after reperfusion the system slips back to normal O_2_ consumption and energy metabolism [14,101]. However, in case of a longer period of ischemia the cell damage gets irreversible and the liver suffers from dysfunction after reperfusion [14,101].

Of course, an experimental validation of our proposed bistable systems behavior needs to be conducted in future. However, our model results do not only provide a possible explanation for the underlying mechanism, it would also offer the possibility to estimate by computational modeling the maximal tolerable ischemia time for steatotic livers. Exceeding this limit during the transplantation process would lead to severe IRI and a considerable increased risk for liver failure. To reach this aim, further quantification of relevant parameters and processes is necessary.

After transplantation, the ischemic injury of a donor organ is aggravated by additional ROS formation in the reperfusion phase, i.e. by reoxygenation [9,12]. In our model, the high state of oxidative stress and LPO reached under hypoxia is a stable one. Therefore, the system persists in this second state also during reoxygenation and a high level of ROS and MDA formation is maintained after reestablishment of the normal O_2_ supply. Although not having explicitly implemented mechanisms of reperfusion injury, our model showed an enhanced ROS formation during reoxygenation. Further processes determining the level of reperfusion injury, such as the initiation of the hepatic inflammatory response [102] can be implemented in future to allow an even more precise prediction of the level of reperfusion injury. Integration of liver damage caused e.g. by neutrophil-mediated oxidative stress [103,104] would surely improve the prediction of the oxidative stress level for steatotic livers.

We are aware, of course, that other processes also influence the degree of IRI in steatotic livers; processes that are not part of the model yet but need to be addressed in future to allow a quantitative prediction of the level of oxidative stress. In the function of fueling the mitochondrial respiratory chain [26], oxidative processes of the carbohydrate metabolism (i.e. glycolysis, TCA cycle) are key factors in ROS formation. Glycolysis is closely linked to the production of ATP, which showed reduced levels in livers after IR [7,29] with consequences for the cell's energy metabolism. In addition, a rapid depletion of hepatic glycogen reserves takes place [26], which influences the level of oxidative damage [105]. Besides the importance of metabolic pathways, their regulation by the cellular signaling network also matters. Of note, metabolic adaptations in response to prolonged hypoxic periods (regulated by the transcription factors HIFs) are not fully integrated into our modeling framework. Studies clearly showed the regulation of hepatic lipid metabolism by these transcription factors [55] and a key role of HIF impairment in steatotic livers as one mechanism for the increased susceptibility [22]. Here, additional modeling effort is needed to include further pathways of oxidative metabolism as well as signaling regarding the adaptive response to transient hypoxia to allow an accurate prediction of the maximal tolerable ischemia duration of steatotic donor organs for transplantation.

Moreover, we focused our modeling effort primarily on the process of LPO (estimated by the TBARS assay) to evaluate IRI, because (1) it is directly linked to the content of FAs and TGs (relevant in the case of steatosis) and (2) the TBARS assay is the most frequently used bioassay to determine the level of oxidative stress in medical studies [40]. Thus, this assay provides the possibility of using already published data for model construction and parameter estimation. However, there are also other indices, which are important to evaluate the degree of IRI, namely the grade of protein oxidation and DNA lesions.

Protein oxidation seems to be also an important driver for the increased susceptibility of steatotic livers [25]. Patients with steatosis exhibit a higher level of protein oxidation, as measured by the liver content of protein carbonyls, compared to a healthy control group [32,106]. Here, protein oxidation leads to structural changes within the cells causing a loss of function. This involves enzymes inactivation, which may contribute to the impairment of AOD (e.g. as shown for the inactivation of the superoxide dismutase by [107]) in a similar way as implemented in our model for the LPO index. Moreover, if the degree of protein oxidation is too large, the repair system for oxidatively modified proteins (namely the 20S-proteasome complex) can become inhibited [108]. Indeed, end products of LPO (e.g. 4HNE) were already reported to inhibit the 20S-proteasome complex [109]. Altogether, these can contribute to the establishment of a second stable state for the protein index or may lead to cell death due to irreversible cell damage. Further modeling effort is necessary to evaluate the outcome of an excessive protein degradation in relation to the repair term during IR.

Oxidative DNA lesions, which means the generation of oxidized bases with high frequency, occur mainly in mitochondria [110] and were reported occasionally in NAFLD [110] and NASH studies [31]. Additionally, there is a correlation between oxidative DNA damage and the grade of inflammation in NASH [31]. In fatty livers, net oxidative DNA damage seems to rely on the efficiency of the repair system rather than on the ROS production rate [110]. Mutations caused by DNA lesions can interfere with the transcription of genes coding for antioxidative enzymes and for respiratory chain components and may alter their expression levels. These promotes mitochondrial and cell dysfunction as well as carcinogenesis. Finally, a high level of oxidative stress leads to genome instability reflecting impairments in the DNA damage repair system [111] and to excess DNA damage, which might initiate cell death. The current model version does not yet include these processes, because of a lack of mechanistic understanding of the correlation between protein and DNA degradation with the hepatic fat content under hypoxic/reoxygenation conditions and how both indices affect the susceptibility of fatty livers to IRI. Overall, further research is necessary to evaluate the interaction between fat content, hypoxia and the level of protein and DNA degradation under IR conditions.

Generally, the grade of steatosis is characterized by the amount of stored TGs in a liver and it is thought to be the key indicator regarding the susceptibility of steatotic livers to IRI. However, lipotoxicity is mainly promoted by FAs [[112], [113], [114]] and FAs, not TGs, promote ROS formation by their oxidative degradation [50,115]. Therefore, we believe that not only the amount of stored TGs is an indicator of the increased susceptibility, but also the intracellular concentration of FAs might be important. In our model, a direct discrimination between the effects of TGs and FAs on ROS formation and LPO is not possible due to the metabolic interrelation between both. Experimental studies however revealed the strong modifying effect of FAs on mitochondrial ROS formation [115] and confirmed that the exposure of liver cells to an increasing amount of FAs does not only lead to an intracellular accumulation of lipids but also to an increased formation of ROS [116]. Generally, FAs can act as key modifiers on the oxidative stress level in three main ways. First, mitochondrial oxidation of FAs fuels the respiratory chain and promotes the formation of ROS [115]. Second, ROS production occurs also directly by the activity of acyl-CoA dehydrogenase [50,51], which is the first enzyme during β-oxidation. And third, FAs are the starting point for oxidative deterioration mediated by free radicals, propagating by free radical chain reactions and ending up in the production of reactive aldehydes [97, 117]. These end products have detrimental effects on liver cells. We clearly see a potential field for further research to answer the question how FAs influence IRI in livers, especially in steatotic livers.

In conclusion, our novel computational model provides a theoretical prediction of a bistable systems behavior triggered by the level of LPO and FAs. This pattern might explain the increased susceptibility of steatotic livers to IRI and provides the possibility to predict the maximal tolerable ischemia time in respect to the severity of hepatic steatosis. In future, we see the potential of computational models in helping to improve the understanding of metabolic adaptations and how this interferes with the FA and TG content in the liver. This would allow a more detailed consideration at which threshold a steatotic liver is still suitable for transplantation and which grade (and type) of steatosis bears a high risk for postoperative liver failure. Such considerations will help to specify selection criteria for organ allocation and, therefore might increase the pool of available donor organs for liver transplantation.

## Appendix A Supplementary data

Supplementary material 1 Image 1

Supplementary material 2 Image 2
