# Supplementary material for: Computational Modeling of Oxidative Stress in Fatty Livers Elucidates the Underlying Mechanism of the Increased Susceptibility to Ischemia/Reperfusion Injury
Source: Comput Struct Biotechnol J. 2018 Nov 1;16:511–22. doi: 10.1016/j.csbj.2018.10.013 (PMC6247397; doi:10.1016/j.csbj.2018.10.013)
Supplement: Supplementary file 1 — Supplementary material 1 [file mmc1.docx]

**Appendix - Supplementary Material**

**Manuscript:** J. Schleicher, U. Dahmen. Computational modeling of oxidative stress in fatty livers elucidates the underlying mechanism of the increased susceptibility to ischemia/reperfusion injury. *Comput Struct Biotechnol J* (2018).

**Appendix A: Detailed model description**

Our computational model contains key factors and their interactions involved in the crosstalk between lipid accumulation and the hepatic stress response to hypoxia. This phenomenological model allows a closer look on the consequences of ischemia and reoxygenation on liver metabolism and reactive oxygen species (ROS) formation. Our intention was not to construct a comprehensive representation of each mechanistic detail of hepatic metabolism. Rather we put emphasize on the simulation of hepatic lipid metabolism and oxidative stress regarding the amount of metabolite supply (fatty acids, FAs, and oxygen, O_2_) via blood.

For model development, we applied a modular approach by starting with the implementation and calibration of a stand-alone submodel of hepatic lipid metabolism. The FA submodel development based on previously models of hepatic lipid accumulation [1, 2]. Subsequently, we added all relevant processes of ROS production and degradation and calibrated the parameters. Finally, the whole model was validated with experimental data from literature. The implementation of equations for the antioxidative defense system (AOD) was based on previously published models [3, 4].

The time unit used for model parameters is hour and has the consequence that the metabolic processes of lipid metabolism proceeds fast to steady state during simulation time. However, most of the parameters in the ROS submodel were guessed due to a lack of experimental data, thus it is unlikely that these modeled processes run at a realistic time scale. Consequently, we designed our model as a qualitative model, which does not allow for quantitative application on the real time scale. More data on parameter values in the ROS submodel are necessary to achieve quantitative model simulations corresponding to real time events.

All model equations were chosen according to current knowledge extracted from literature and, thus, provide a representation of the present understanding of FA and ROS metabolism in the liver. In this supplement, we provide all model details including equations, parameter settings and used calibration data. Concentrations of substrates, metabolites and products are in mM (in regard to 1l of liver volume or 1l of blood, respectively). If experimental data reported in literature differ in units, we used the following relations to transform in mM. We used a mean liver density of 1050g/l [5, 6] to transform values from liver weight to liver volume. We assumed a mean hepatocyte number of 139 million cells/g liver [6] and used the assumption of 0.657 mg protein/10^6^ cells [6]. The molecular weight of triglycerides (TGs) was assumed to be around 850 g/mol according to Farquhar et al. [7].

The model was implemented, calibrated and analyzed in the software R [8] using the packages ‘deSolve’ [9] and ‘FME’ [10].

*A1 Mathematical model of hepatic lipid metabolism*

FAs are delivered to liver cells (hepatocytes) by blood flow through the hepatic blood vessels (called sinusoids). Total concentration of FAs in human plasma are at normal physiological conditions between 0.1 mM and 0.4 mM [11, 12], with up to more than 1 mM in patients with nonalcoholic fatty liver disease [12-14]. Therefore, a range from 0.1 mM to 1.4 mM of plasma FA concentration (parameter *[FA]_blood_*) was used in the model to simulate hepatic metabolism under a normal diet up to a chronic high‑fat diet (HFD), respectively. Uptake of FAs from blood into hepatocytes (**Equation 1**) depends on plasma FA concentration [15] and the stored TG content. The uptake rate is almost linear with the plasma FA concentration [11, 16], but shows a saturation [15], in our model determined by a certain amount of stored TGs. This dependence on TG content assumes that FA uptake saturates due to a saturation of intracellular lipid metabolism [11]. Thus, the TG content refers here as a marker of saturation of intracellular lipid metabolism and, for this purpose, a specific threshold (parameter *[TG]_ss_*) was implemented.


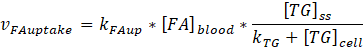
 . **Eq. 1**

O_2_ is mainly delivered via arterial blood to the hepatocytes by approximately 200 ml O_2_ per liter of blood. With 22400 ml O_2_/mol, this corresponds to about 8.9 mmol O_2_/l_blood_ for normal tissue perfusion. This value was used for simulations under normoxic conditions (parameter *[O2]_blood_*). During ischemia, hepatocytes suffer from O_2_ paucity, which leads to cellular hypoxia. To simulate hypoxic conditions, the *[O2]_blood_* value was reduced to 0.001 mM according to literature sources reporting O_2_ concentrations under hypoxia between 0.003 mM [17] and 0.0001 mM [18]. Michaelis-Menten kinetics is used to simulate cellular O_2_ uptake (**Equation 2**). Here, maximal O_2_ uptake measured in hepatocyte cultures can be used as an approximation for maximal velocity of the uptake process (parameter *v_max_^O2up^*; [19]). The Michaelis-Menten constant *K_M_^O2up^* for O_2_ affinity is supposed to be very low [19].


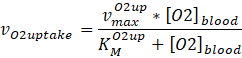
. **Eq. 2**

Mitochondrial oxidation of FAs is represented by two-substrate Michaelis-Menten kinetics (**Equation 3a**), following Reed et al. [4], and depends on both cellular FA and O_2_ concentrations:


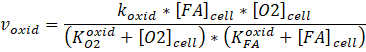
. **Eq. 3a**

O_2_ is consumed also by other processes than mitochondrial FA oxidation, e.g. glucose oxidation. Therefore, we implemented simple mass-action kinetics (**Equation 4**) to account for these processes in a rather unspecific way:


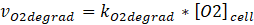
. **Eq. 4**

Synthesis of TGs depends mainly on substrate concentration within the hepatocytes [20]. Therefore, in our model intracellular FA concentration (state variable *[FA]_cell_*) is set as the major driver of TG synthesis (**Equation 5**), thus its rate can be approximated by a linear relationship [21] without saturation [22]. The backward rate of TG synthesis is necessary for depletion of the intracellular TG pool but can be assumed to be low at physiological conditions. In support, experimental studies reveal that only a minor part of FAs from the TG pool is directed to oxidation [23].


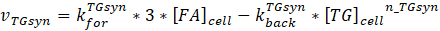
. **Eq. 5**

The export or secretion of TGs follows a Michaelis-Menten kinetics (**Equation 6**). This choice of equation type is based on the observation that TG export reaches a plateau with increasing FA supply [22, 24]:


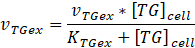
. **Eq. 6**

*De novo* lipogenesis (DNL), i.e. the production of new FAs from acetyl-CoA, may also contribute to TG synthesis and accumulation under certain conditions (see discussion in [2]). Particularly, a high-carbohydrate diet enhances hepatic DNL, whereby causing aberrant TG accumulation [25]. However, the contribution of this process in our considered setting is assumed to be only a minor one [23], because we focus on a HFD with an assumed normal content of carbohydrates. In support, the excess supply of FAs does, in fact, inhibit the process of DNL [26]. Generally, with our model we focus on the intermingling of FA metabolism and ROS production neglecting the effects of carbohydrate metabolism.

In sum, the following system of ordinary differential equations of three state variables can be deduced:


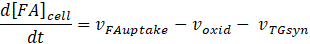
, **Eq. 7**


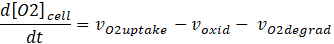
, **Eq. 8a**


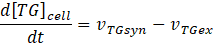
. **Eq. 9**

*A2 Mathematical model of ROS formation linked to hepatic lipid metabolism*

Here, we shortly describe the submodel of ROS production and detoxification (i.e. the hepatic AOD), which is linked to the lipid submodel described above. We focused on hydrogen peroxide (H_2_O_2_) as ROS, because it is more stable than the superoxide anion O_2_^-^. Additionally, O_2_^-^, after produced by the electron transport chain, is directly transformed to H_2_O_2_.

The production of H_2_O_2_ (**Equation 10**) is coupled to the concentration of intracellular O_2_ (state variable *[O2]_cell_*) and the rate of mitochondrial FA oxidation (*v_oxid_*), thus encompassing the production of H_2_O_2_ by the activity of the mitochondrial respiration rushed by FA β-oxidation. Here, O_2_ acts as an electron acceptor. The link between FA oxidation and ROS production is justified by the observation that FA supplementation enhances mitochondrial ROS release [27]. ROS production in the mitochondria occurs because O_2_ is not completely reduced to water, which is unavoidable and takes place naturally at a low basal rate [28].


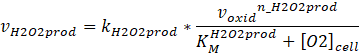
 . **Eq. 10**

The AOD for H_2_O_2_ is mainly composed of two enzymes: catalase (CAT) and glutathione peroxidase (GPx). Both enzymes are features in past modeling approaches, so that we used previously established equations and parameter values to simulate hepatic H_2_O_2_ neutralization.

The equation of CAT (**Equation 11**) is based on Cortassa et al. [3]. We modified this equation by adding an inhibitory term for the effect of lipid peroxidation (LPO; represented by the concentration of MDA, explanation see below) on enzyme activity. The detoxification capability mediated by CAT (and also by GPx, see below) raises with an increasing level of oxidative stress (i.e. increased production of H_2_O_2_), however at a certain level both enzymes become depleted and the detoxification capability drops down [29-31]. This impaired detoxification capability is based on the oxidative damage to cellular lipids: (1) the products of LPO bind to the active site of antioxidant enzymes and, thus inhibit their activity [29], and (2) the peroxidation of membrane lipids (particularly of the mitochondrial membrane) leads to glutathione (GSH) pool reduction [32].

$$v_{CAT}=\frac{2*k_{CAT}*E_{CAT}*\left[ H2O2 \right]_{cell}*e^{-k_{inh}*\left[ H2O2 \right]_{cell}}}{1+\left( \frac{\left[ MDA \right]_{cell}}{K_{i}^{CAT}} \right)^{n\_CAT}}$$

 . **Eq. 11**

The activity of GPx (**Equation 12**) was modeled according to Cortassa et al. [3] and Reed et al. [4], but contrary to previous models we did not specifically simulate the change in hepatic GSH concentration but used a fixed mean value reported by Starkov [33]. Similar to the equation for CAT activity, we added an inhibitory term for the effect of LPO (represented by the MDA concentration) on GPx activity (explanation see above).

$v_{GPx}=\frac{v_{max}^{GPx}*\left[ H2O2 \right]_{cell}*\left[ GSH \right]}{\left( K_{M}^{GSH}+\left[ GSH \right] \right)*\left( K_{M}^{H2O2}+\left[ H2O2 \right]_{cell} \right)*\left( 1+\left( \frac{\left[ MDA \right]_{cell}}{K_{i}^{GPx}} \right)^{n\_GPx} \right)}$. **Eq. 12**

The level of H_2_O_2_ determines the production rate of the hydroxyl radical (OH^*^; **Equation 13**), which is the most important ROS in regard to intracellular damage due to its high reactivity [32]. OH^*^ is highly reactive, thus very short-living, so that we decided to calculate its production rate but do not simulate its specific intracellular concentration. There seems to be no direct defense mechanism against OH^*^ [32].


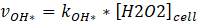
 . **Eq. 13**

OH^*^ oxidizes intracellular lipids, thereby initiating LPO, which leads to the production of cytotoxic intermediates such as malondialdehyde (MDA) and 4-hydroxynonenal (4HNE) [34]. In our model, the level of LPO is represented by the concentration of MDA (state variable *[MDA]_cell_*), which is determined by the rate of OH^*^ production (*v_OH*_*) and the concentration of stored lipids (state variable *[TG]_cell_*) (**Equation 14**). Integration of the hepatic TG concentration into the equation rate is based on the observation that the degree of LPO correlates with the grade of steatosis [35, 36]. The explosion in MDA concentration is prevented by cellular repair or protection mechanisms (i.e. antioxidants such as vitamin E; [37]). We assumed linear kinetics for the repair mechanism (**Equation 15**) representing the enzymatic metabolization of MDA [37].

$$v_{MDA}=k_{MDA}*v_{OH*}*{\left[ TG \right]_{cell}}^{n\_MDA}$$

 , **Eq. 14**

$v_{MDArep}=k_{MDArep}*\left[ MDA \right]_{cell}$. **Eq. 15**

Finally, to simulate the effect of hypoxia on FA metabolism, we modulate the equation of FA oxidation (**Equation 3a**) by an additional term. The oxidation rate of FAs is influenced under hypoxic conditions by the expression of the hypoxia-inducible factors (HIFs; [38]), which mediate metabolic adaptations under O_2_ paucity [39]. A decreasing O_2_ gradient leads to a switch-like response of HIF activation with a plateau at very low O_2_ levels [40]. To account for this effect in our modeling framework, we adjusted the equation of FA oxidation (**Equation 3a**) by adding a sigmoidal term depending on intracellular O_2_ concentration (state variable *[O2]_cell_*):


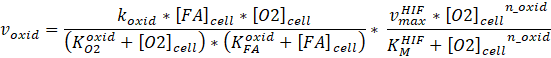
 . **Eq. 3b**

The system of ordinary differential equations presented above (**Equation 7-9**) can be extended by two oxidative stress specific state variables and a modified equation for the intracellular O_2_ concentration:

$$\frac{d\left[ O2 \right]_{cell}}{dt}=v_{O2uptake}-v_{oxid}- v_{O2degrad}-v_{H2O2prod}$$

 , **Eq. 8b**

$$\frac{d[{H2O2]}_{cell}}{dt}=v_{H2O2prod}-v_{CAT}-v_{GPx}-v_{OH*}$$

, **Eq. 17**

$$\frac{d{[MDA]}_{cell}}{dt}=v_{MDA}-v_{MDArep}$$

. **Eq.18**

**Table A1.** Parameter values and their sources; for further details see below section “A2 Calibration and parameter estimation”. In the following, we list all parameters and their values used in the mathematical model.

Abbr.: CAT = catalase, dl = dimensionless, FA = fatty acid, GPx = glutathione peroxidase, GSH = reduced glutathione, HIF = hypoxia-inducible factor, H_2_O_2_ = hydrogen peroxide, LPO = lipid peroxidation, O_2_ = oxygen, TG = triglycerides.

| **Parameter** | **Value (unit)** | **Reference** |
| --- | --- | --- |
| **Lipid metabolism submodel** | | |
| FA_blood_ | 0.1 mM - 1.4 mM | range of plasma FA concentrations measured in humans [11-14] |
| k_FAup_ | 35.937 h^-1^ | calculated from Heimberg et al. [24] |
| k_TG_ | 0.08936555 mM | fitted to data from Heimberg et al. [24] and van Harken et al. [41] |
| [TG]_ss_ | 6.654673 mM | fitted to data from Heimberg et al. [24] and van Harken et al. [41] |
| [O2]_blood_ | 8.9 mM (normoxia, reoxygenation) / 0.001 mM (hypoxia) | calculated from normal plasma values (humans), see detailed model description (Appendix A1) |
| v_max_^O2up^ | 143.85 mM/h | calculated from maximal O_2_ uptake in Matsumura et al. [42] |
| K_M_^O2up^ | 0.001 mM | Wagner et al. [19] |
| k_oxid_ | 25.2 mM/h | estimated from Mannaerts et al. [43] |
| K_O2_^oxid^ | 0.001 mM | estimated to be very low (due to high affinity for O_2_) |
| K_FA_^oxid^ | 0.122 mM | Michaelis-Menten constant for carnitine palmitoyltransferase I [44] |
| k_O2degrad_ | 500 h^-1^ | estimated (to fit reported intracellular O_2_ concentrations [45]) |
| k_for_^TGsyn^ | 9.972427 h^-1^ | fitted to data from Heimberg et al. [24] and van Harken et al. [41] |
| k_back_^TGsyn^ | 0.00002712017 h^-1^ | fitted to data from Heimberg et al. [24] and van Harken et al. [41] |
| n_TGsyn | 4.942853 (dl) | fitted to data from Heimberg et al. [24] and van Harken et al. [41] |
| v_TGex_ | 1.55 mM/h | estimated from Heimberg et al. [24] |
| K_TGex_ | 6.061116 mM | fitted to data from Heimberg et al. [24] and van Harken et al. [41] |
| **Reactive oxygen species submodel** | | |
| k_H2O2prod_ | 0.396 mM | calculated from Adimora et al. [46] |
| n_H2O2prod | 0.65 (dl) | estimated |
| K_M_^H2O2prod^ | 0.08 mM | estimated |
| k_CAT_ | 5400000 1/mM*h | Tovmasyan et al. [47] |
| E_CAT_ | 0.0012 mM | Gonzalez-Flecha et al. [45] |
| k_inh_ | 0.05 1/mM | Gauthier et al. [48] |
| K_i_^CAT^ | 0.1 mM | estimated |
| n_CAT | 4 (dl) | estimated |
| v_max_^GPx^ | 4.5 mM/h | Reed et al. [4] |
| [GSH] | 8 mM | estimated from Starkov [33] |
| K_M_^GSH^ | 1.3 mM | Reed et al. [4] |
| K_M_^H2O2^ | 0.00009 mM | Reed et al. [4] |
| K_i_^GPx^ | 0.07 mM | estimated |
| n_GPx | 4 (dl) | estimated |
| k_OH*_ | 10 h^-1^ | estimated |
| k_MDA_ | 0.3 mM^-1^ | estimated |
| n_MDA | 0.1 (dl) | estimated |
| k_MDArep_ | 0.068 h^-1^ | estimated |
| v_max_^HIF^ | 1 mM/h | estimated |
| n_oxid | 2.5 (dl) | estimated |
| K_M_^HIF^ | 0.0005 mM | estimated |

**Appendix B: Calibration and parameter estimation**

*B1 Lipid submodel*

Most values of the kinetic parameters in the lipid submodel were extracted from current literature (see Table A1). However, some kinetic parameters (e.g. in TG synthesis) are unknown, thus we performed a parameter fitting procedure. In principle, to find reasonable values for unknown parameters, parameter values were adjusted until a good agreement with reported (steady state) substrate concentrations or metabolic rates were achieved. This was handled in the following way:

(1) Normal intracellular concentration of O_2_ in hepatocytes was reported to be around 0.22 mM under physiological conditions [45]. The parameter in the rate of O_2_ degradation (*k_O2degrad_*) was adjusted until the cellular O_2_ concentration in the model reaches this value for a simulation under low plasma FA supply (parameter *[FA]_blood_* = 0.1 mM).

(2) Parameter values of TG synthesis were estimated by using the function *modfit* from the ‘FME’ package (see detailed description below). The procedure was done by fitting the simulated steady-state concentration of TGs (over the whole range of *FA_blood_*) to data extracted from Heimberg et al. [24] and van Harken et al. [41]. Both papers reported hepatic TG concentrations as a function of the FA concentration in the medium. The result of the best-fit parameter set is shown in Fig. B1.

(3) Parameter values of TG export were obtained with the *modfit* function from the ‘FME’ package (see below) by fitting the rate of TG export (simulated over the whole range of *[FA]_blood_*) to data extracted from Heimberg et al. [24]. This paper reported the rate of TG secretion as a function of the declining FA concentration in the medium. The result of the best-fit parameter set is shown in Fig. B1.


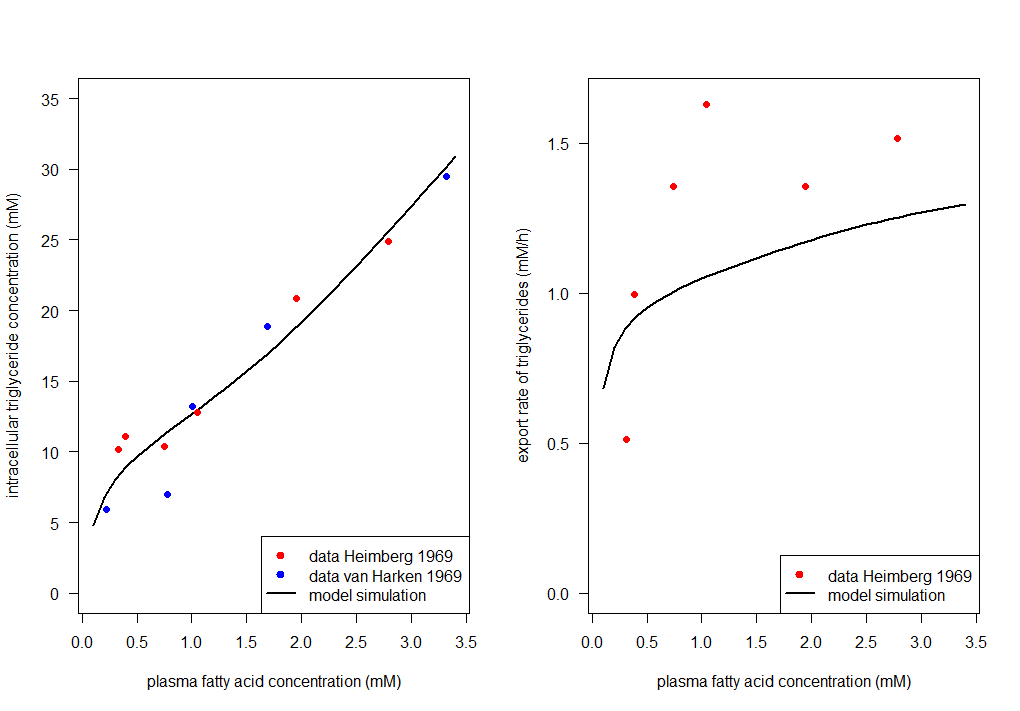


**Figure B1. Parameter estimation of triglyceride (TG) synthesis and export.** Simulation output was fitted to experimental data obtained from Heimberg et al. [24] and van Harken et al. [41] by using the function *modfit*.

The fitting procedure of parameter values to reported experimental data was performed in accordance to the inverse modeling approach described in Soetaert and Petzoldt [10] with the ‘FME’ package. All other parameter values, which are known from literature, were set fixed. Here, we give a short description of the steps how we performed parameter estimation of the remaining, unknown parameters:

(1) First, for all parameters we performed an identifiability analysis with the function *collin* of the ‘FME’ package. This analysis allows finding parameter sets, which can be estimated with good precision from the available experimental data points. Of note, parameter identifiability is the first step to ensure getting a reasonable guess for the unknown parameters. Specifically, the *collin* function estimates the approximate linear dependence of all possible combinations of parameter sets [10]. Here, sets with large dependencies (collinearity value >20) are not identifiable by the used experimental data. According to Soetaert and Petzoldt [10], collinearity γ is calculated by


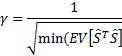
 , where *EV* is the eigenvalues and $\hat{S}$ contains the sensitivity matrix of the combinations of parameter sets (for *i* output variables and *j* parameters) and was calculated by


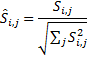
 .

(2) Then, the identifiable parameter sets were fitted to the experimental data by the application of an optimization routine. The function *modFit* from the ‘FME’ package fits the model parameters to data by minimizing residuals, i.e. the deviation of simulated values from experimental values. Here, the *modCost* function in the ‘FME’ package allows an automatic calculation of weighted residuals, and the sum of squared residuals. According to Soetaert and Petzoldt [10], weighted and scaled residuals are calculated as:


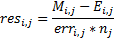
, with *i* observed data points of *j* variables, *M_i,j_* are modeled values and *O_i,j_* are observed, experimentally determined values, *err_i,j_* is a weighing factor and *n_j_* is the number of data points of each variable, which can be used as scaling factor. The *modCost* function was applied to calculate the residuals between our model simulation results and the data extracted from Heimberg et al. [24] and van Harken et al. [41]. The function *modFit*, then, searches for the parameter values that minimize these “costs”. The default procedure (Levenberg-Marquardt algorithm) of *modfit* was applied.

*B2 ROS submodel (linked to the lipid submodel)*

Parameter values in the ROS submodel were also extracted from current literature (see Table A1). However, most of the kinetic parameters (e.g. for LPO processes) are unknown , thus we were forced to make a reasonable guess. In doing this, we adjusted parameter values until a good agreement with the following observations and measurements reported in literature were achieved:

(1) The concentration of H_2_O_2_ in hepatocytes is around 9 x 10^-5^ mM under non-pathological conditions [45] and the rate of H_2_O_2_ production is in the range of 0.1764 mM/h - 0.504 mM/h [17]. We adjusted model parameters to reach these values at a low plasma FA concentration (parameter *[FA]_blood_*).

(2) LPO-mediated cell injury is represented in our model by the concentration of MDA. In general, the concentration of MDA is widely used as a marker of hepatic oxidative damage mediated by LPO [49]. The physiological level of MDA in human liver samples is around 0.00365 mM [50]. In our model, parameters were adjusted to reach this value at low *[FA]_blood_* values.

(3) The parameters of the effect of MDA on CAT and GPx were chosen so that under low to medium values of *[FA]_blood_* the GPx activity is higher than the CAT activity and no inhibition of activity occurs by MDA. At higher *[FA]_blood_* values GPx gets inhibited strongly, thus at pathological values CAT activity is more important for H_2_O_2_ detoxification. This is based on the observation of Antunes et al. [51]: The importance of GPx for H_2_O_2_ detoxification is at physiological normal conditions greater than the CAT activity. However, CAT gets more important at H_2_O_2_ concentrations that overcome the GPx system.

**Appendix C: Robustness analysis**

Considering standard deviations in parameter values would reveal the robustness of model predictions. To show the effects of an assumed 10% standard deviation in parameter values, we conducted 100 runs with different parameter values. Before a run, for each parameter a value was drawn randomly from a normal distribution with the value from the original model as mean and a 10% standard deviation. The model was run under normoxia for a range of MDA concentrations like the runs reported for the original parameter values in the results section 3.2 in the manuscript. The results of the first 10 runs are presented in the manuscript in Fig. 4, whereas the results of the remaining runs are reported in Fig. C1-C9. A summary is provided in Fig. C10.


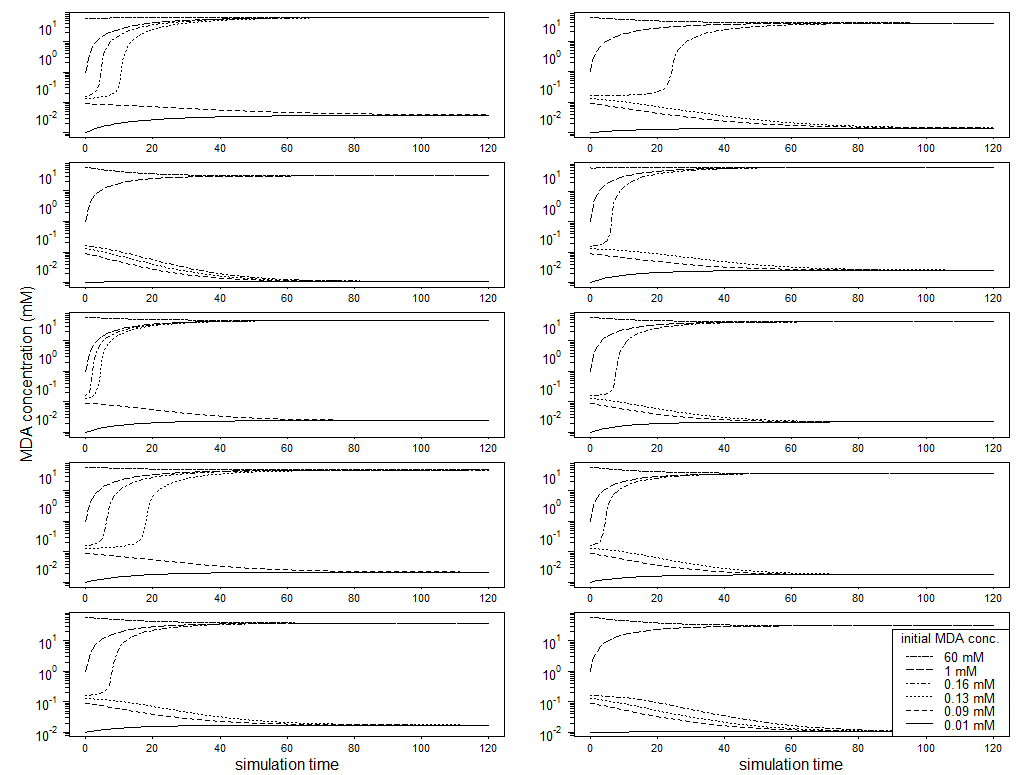


**Figure C1:** 10 out of 100 runs with randomly drawn parameter values.


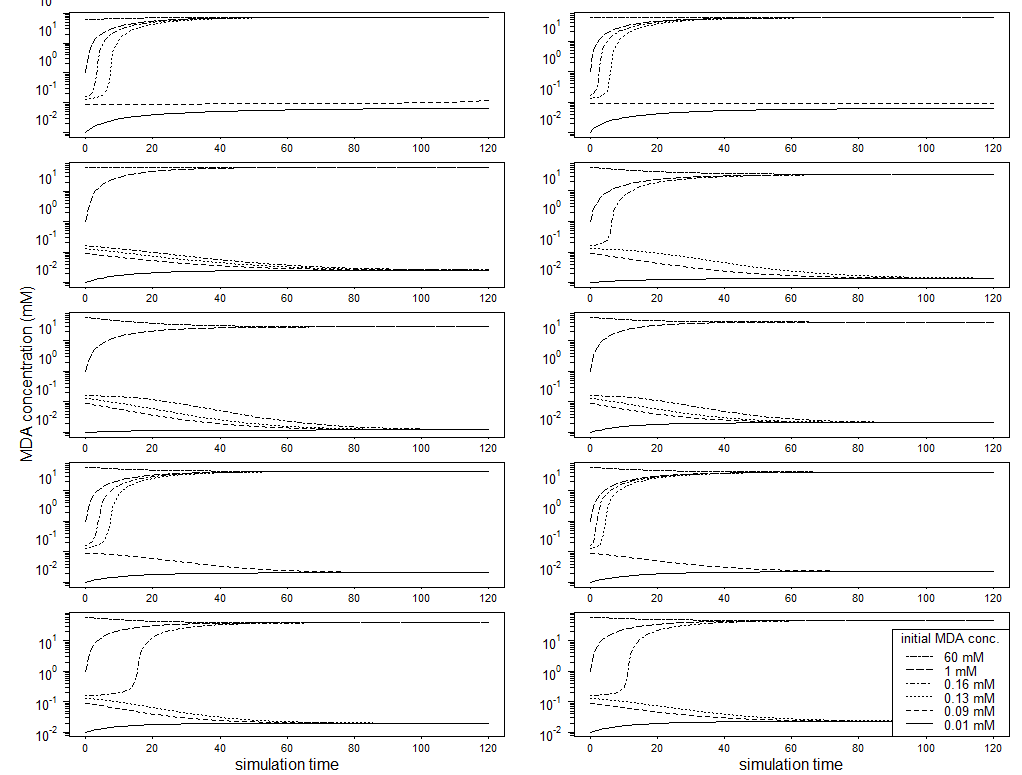


**Figure C2:** 10 out of 100 runs with randomly drawn parameter values.


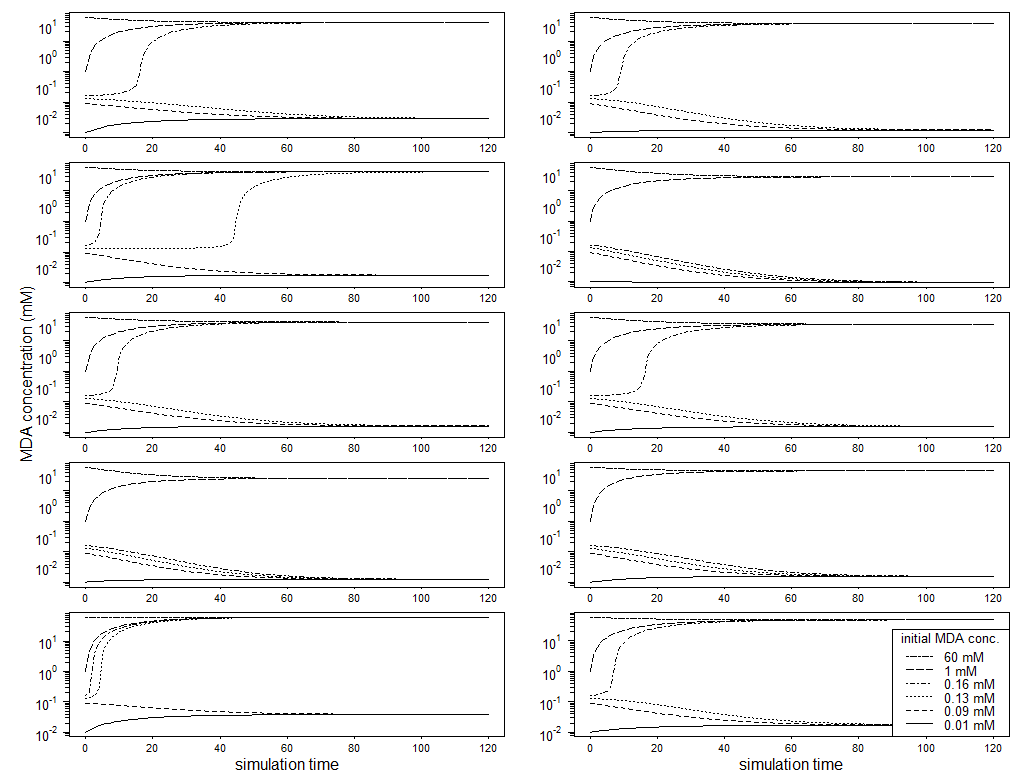


**Figure C3:** 10 out of 100 runs with randomly drawn parameter values.


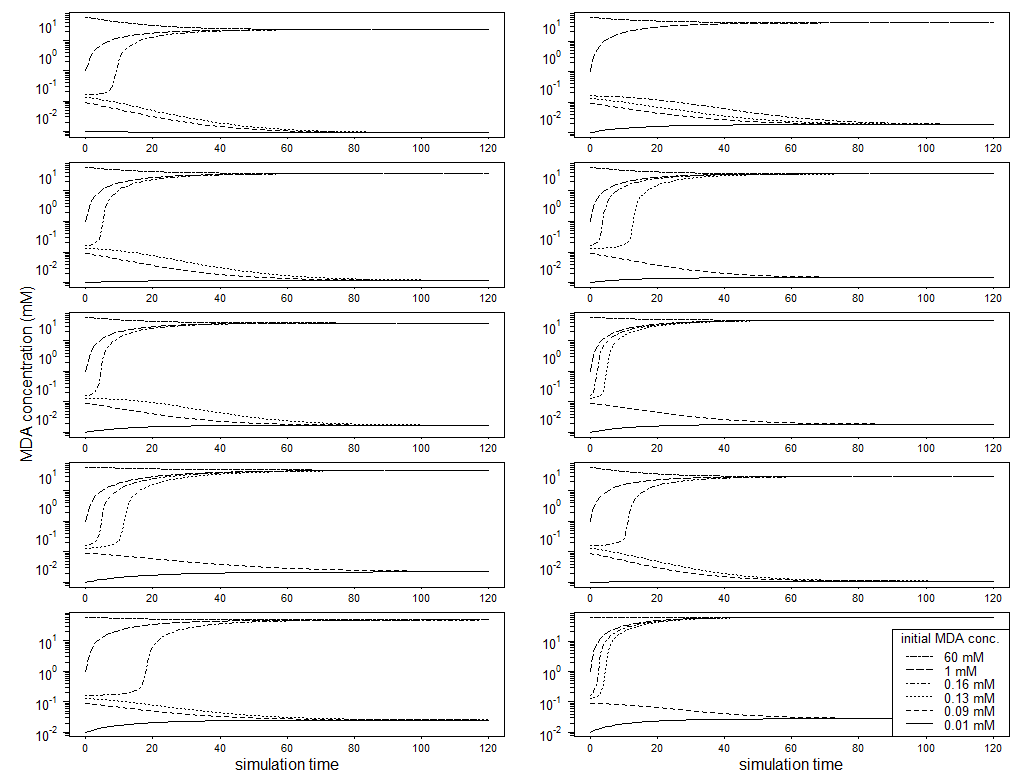


**Figure C4:** 10 out of 100 runs with randomly drawn parameter values.


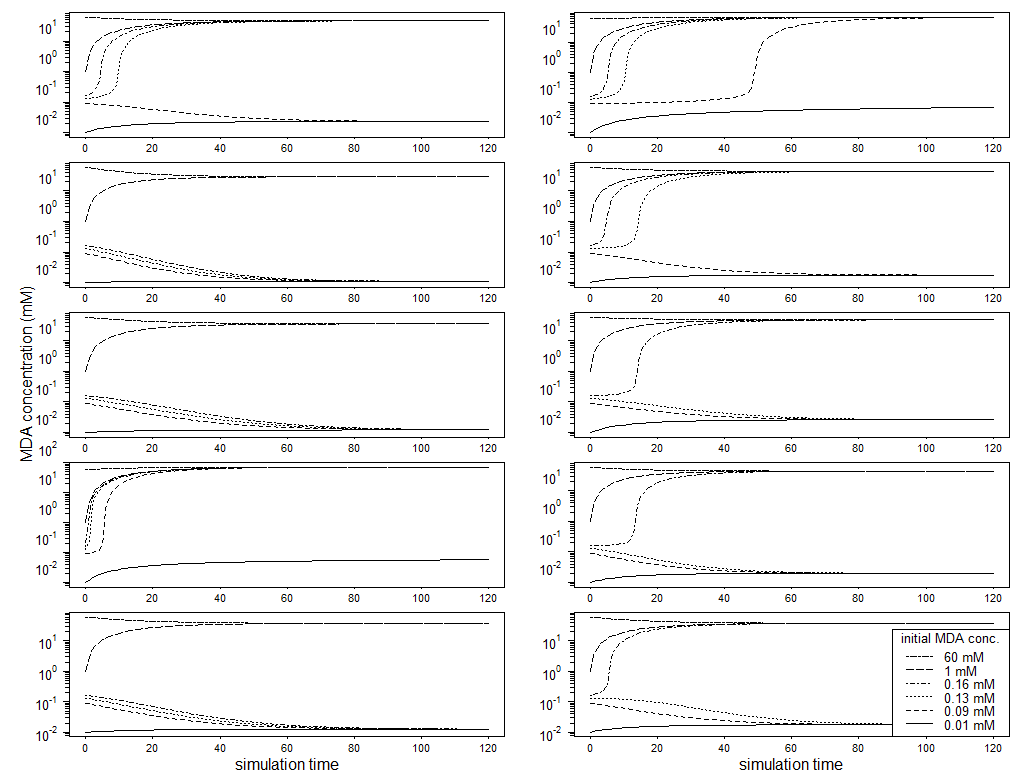


**Figure C5:** 10 out of 100 runs with randomly drawn parameter values.


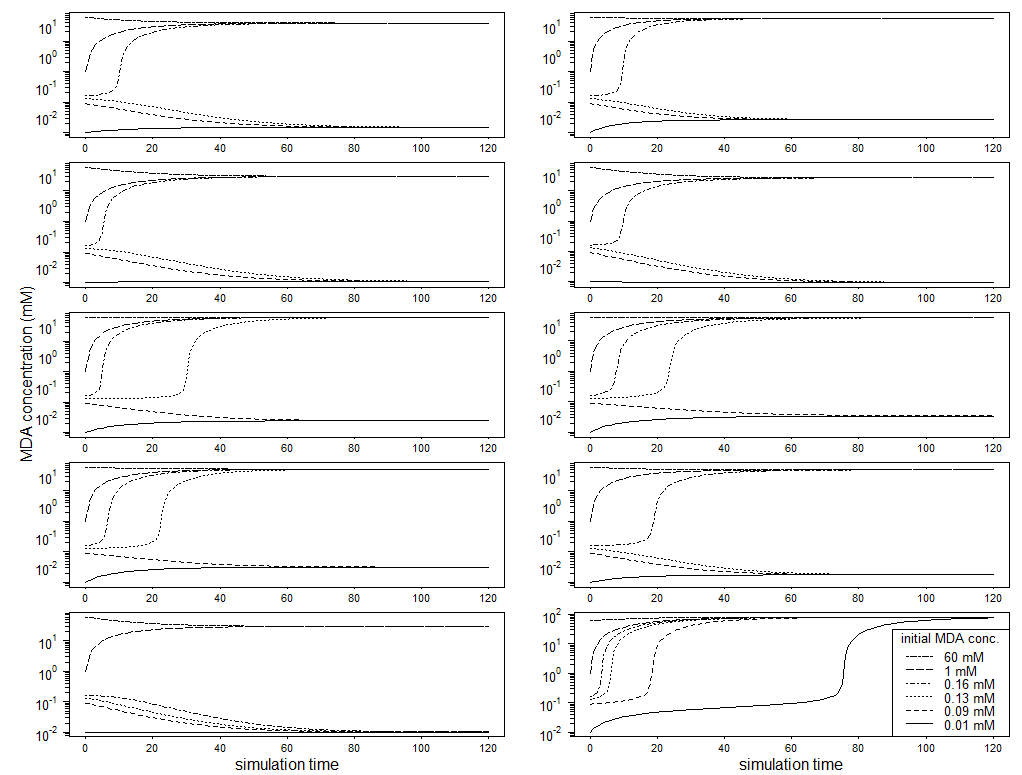


**Figure C6:** 10 out of 100 runs with randomly drawn parameter values.


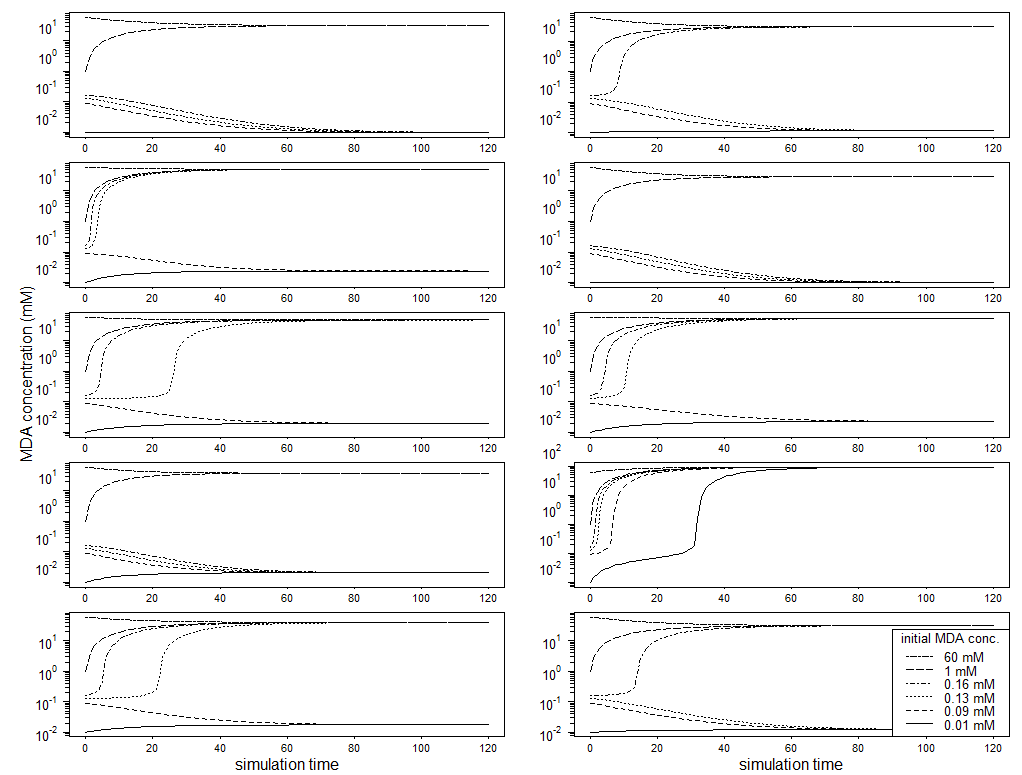


**Figure C7:** 10 out of 100 runs with randomly drawn parameter values.


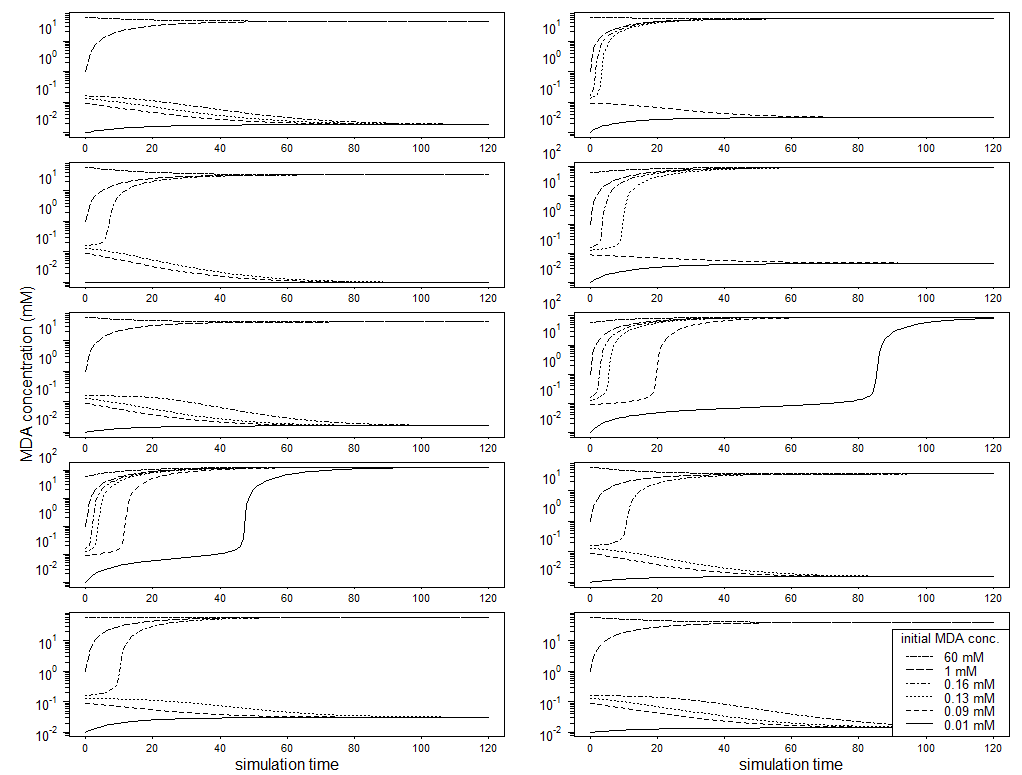


**Figure C8:** 10 out of 100 runs with randomly drawn parameter values.


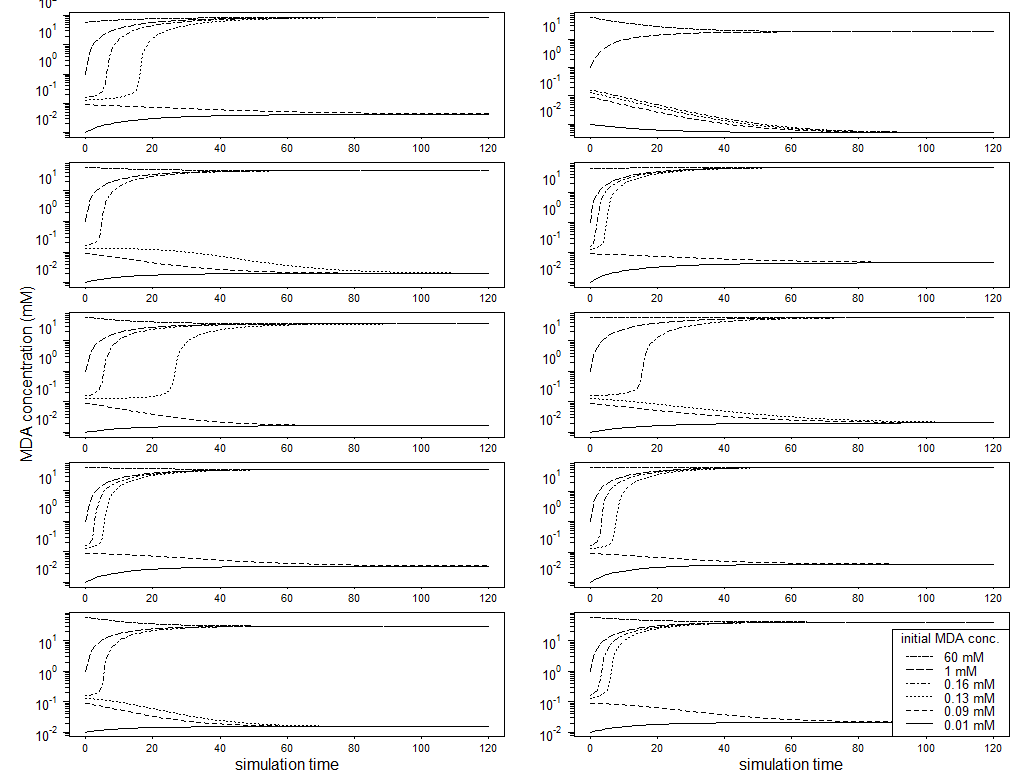


**Figure C9:** 10 out of 100 runs with randomly drawn parameter values.


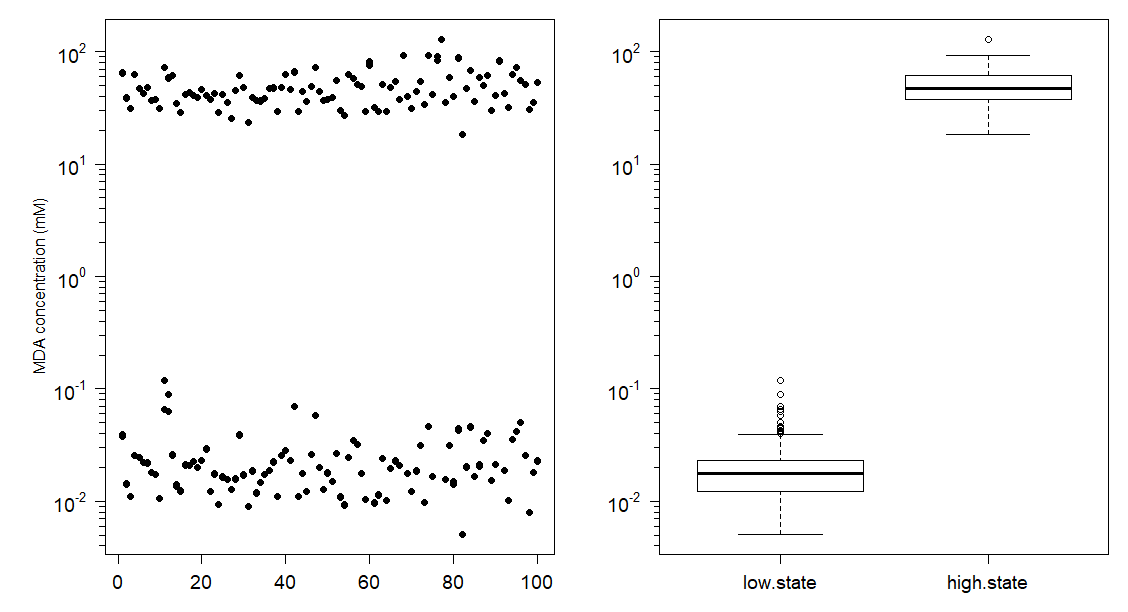


**Figure C10:** Summary of all 100 runs, each with randomly drawn parameter values.

**Appendix D:** Code of the mathematical model is provided as a separate R file: *AppendixD_ModelCodeForR.R*

**Literature**

1. J. Schleicher, R. Guthke, U. Dahmen, O. Dirsch, H.G. Holzhuetter, S. Schuster **A theoretical study of lipid accumulation in the liver-implications for nonalcoholic fatty liver disease** Biochim Biophys Acta, 1841 (2014), pp. 62-69

2. J. Schleicher, U. Dahmen, R. Guthke, S. Schuster **Zonation of hepatic fat accumulation: insights from mathematical modelling of nutrient gradients and fatty acid uptake** J R Soc Interface, 14 (2017)

3. S. Cortassa, M.A. Aon, R.L. Winslow, B. O'rourke **A mitochondrial oscillator dependent on reactive oxygen species** Biophys J, 87 (2004), pp. 2060-2073

4. M.C. Reed, R.L. Thomas, J. Pavisic, S.J. James, C.M. Ulrich, H.F. Nijhout **A mathematical model of glutathione metabolism** Theor Biol Med Model, 5 (2008), pp. 8

5. B.A. Overmoyer, C.E. Mclaren, G.M. Brittenham **Uniformity of liver density and nonheme (storage) iron distribution** Arch Pathol Lab Med, 111 (1987), pp. 549-554

6. A.-K. Sohlenius-Sternbeck **Determination of the hepatocellularity number for human, dog, rabbit, rat and mouse livers from protein concentration measurements** Toxicol In Vitro, 20 (2006), pp. 1582-1586

7. J.W. Farquhar, R.C. Gross, R.M. Wagner, G.M. Reaven **Validation of an incompletely coupled two-compartment nonrecycling catenary model for turnover of liver and plasma triglyceride in man** J Lipid Res, 6 (1965), pp. 119-134

8. R. Core-Team, **R: A language and environment for statistical computing**. 2017, Vienna, Austria, 10.1007/978-3-540-74686-7

9. K. Soetaert, T. Petzoldt, W. Setzer **Solving differential equations in R: Package deSolve.** J Statist Software, 33 (2010), pp. 1-25

10. K. Soetaert, T. Petzoldt **Inverse modelling, sensitivity and Monte Carlo analysis in R using package FME** J Statist Software, 33 (2010), pp. 1-28

11. J.F. Glatz, J.J. Luiken, A. Bonen **Membrane fatty acid transporters as regulators of lipid metabolism: implications for metabolic disease** Physiol Rev, 90 (2010), pp. 367-417

12. J. Zhang, Y. Zhao, C. Xu, Y. Hong, H. Lu, J. Wu*, et al.* **Association between serum free fatty acid levels and nonalcoholic fatty liver disease: a cross-sectional study** Sci Rep, 4 (2014), pp. 5832

13. S.A. Abdelmagid, S.E. Clarke, D.E. Nielsen, A. Badawi, A. El-Sohemy, D.M. Mutch*, et al.* **Comprehensive profiling of plasma fatty acid concentrations in young healthy Canadian adults** PLoS One, 10 (2015), pp. e0116195

14. R. Gambino, E. Bugianesi, C. Rosso, L. Mezzabotta, S. Pinach, N. Alemanno*, et al.* **Different serum free fatty acid profiles in NAFLD subjects and healthy controls after oral fat load** Int J Mol Sci, 17 (2016), pp. 479

15. M.W. Bradbury **Lipid metabolism and liver inflammation. I. Hepatic fatty acid uptake: possible role in steatosis** Am J Physiol-Endoc M, 290 (2006), pp. G194-198

16. P.D. Berk **Regulatable fatty acid transport mechanisms are central to the pathophysiology of obesity, fatty liver, and metabolic syndrome** Hepatology, 48 (2008), pp. 1362-1376

17. P. Jezek, L. Hlavata **Mitochondria in homeostasis of reactive oxygen species in cell, tissues, and organism** Int J Biochem Cell Biol, 37 (2005), pp. 2478-2503

18. D.L. Hoffman, J.D. Salter, P.S. Brookes **Response of mitochondrial reactive oxygen species generation to steady-state oxygen tension: Implications for hypoxic cell signaling** Am J Physiol Heart Circ Physiol, 292 (2007), pp. H101-108

19. B.A. Wagner, S. Venkataraman, G.R. Buettner **The rate of oxygen utilization by cells** Free Radic Biol Med, 51 (2011), pp. 700-712

20. D.F. Vatner, S.K. Majumdar, N. Kumashiro, M.C. Petersen, Y. Rahimi, A.K. Gattu*, et al.* **Insulin-independent regulation of hepatic triglyceride synthesis by fatty acids** Proc Natl Acad Sci U S A, 112 (2015), pp. 1143-1148

21. H.K. Stals, W. Top, P.E. Declercq **Regulation of triacylglycerol synthesis in permeabilized rat hepatocytes. Role of fatty acid concentration and diacylglycerol acyltransferase** FEBS letters, 343 (1994), pp. 99-102

22. W. Patsch, T. Tamai, G. Schonfeld **Effect of fatty acids on lipid and apoprotein secretion and association in hepatocyte cultures** J Clin Invest, 72 (1983), pp. 371-378

23. G.F. Gibbons, K. Islam, R.J. Pease **Mobilisation of triacylglycerol stores** Biochim Biophys Acta, 1483 (2000), pp. 37-57

24. M. Heimberg, I. Weinstein, M. Kohout **The effects of glucagon, dibutyryl cyclic adenosine 3',5'-monophosphate, and concentration of free fatty acid on hepatic lipid metabolism** J Biol Chem, 244 (1969), pp. 5131-5139

25. F. Diraison, P. Moulin, M. Beylot **Contribution of hepatic de novo lipogenesis and reesterification of plasma non esterified fatty acids to plasma triglyceride synthesis during non-alcoholic fatty liver disease** Diabetes Metab, 29 (2003), pp. 478-485

26. A. Ferramosca, A. Conte, F. Damiano, L. Siculella, V. Zara **Differential effects of high-carbohydrate and high-fat diets on hepatic lipogenesis in rats** Eur J Nutr, 53 (2013), pp. 1103-1114

27. A.R. Cardoso, P.A. Kakimoto, A.J. Kowaltowski **Diet-sensitive sources of reactive oxygen species in liver mitochondria: role of very long chain acyl-CoA dehydrogenases** PLoS One, 8 (2013), pp. e77088

28. R. Mittler **ROS are good** Trends Plant Sci, 22 (2017), pp. 11-19

29. R. Polavarapu, D.R. Spitz, J.E. Sim, M.H. Follansbee, L.W. Oberley, A. Rahemtulla*, et al.* **Increased lipid peroxidation and impaired antioxidant enzyme function is associated with pathological liver injury in experimental alcoholic liver disease in rats fed diets high in corn oil and fish oil** Hepatology, 27 (1998), pp. 1317-1323

30. G. Perlemuter, A. Davit-Spraul, C. Cosson, M. Conti, A. Bigorgne, V. Paradis*, et al.* **Increase in liver antioxidant enzyme activities in non-alcoholic fatty liver disease** Liver Int, 25 (2005), pp. 946-953

31. T. Saricam, B. Kircali, T. Koken **Assessment of lipid peroxidation and antioxidant capacity in non-alcoholic fatty liver disease** Turk J Gastroenterol, 16 (2005), pp. 65-70

32. C. Borza, D. Muntean, C. Dehelean, G. Savoiu, C. Serban, G. Simu*, et al.*, **Oxidative stress and lipid peroxidation - A lipid metabolism dysfunction**, in *Lipid Metabolism*, R. Baez Valenzuela, Editor. 2013, INTECH

33. A.A. Starkov **The role of mitochondria in reactive oxygen species metabolism and signaling** Ann N Y Acad Sci, 1147 (2008), pp. 37-52

34. A.P. Rolo, J.S. Teodoro, C.M. Palmeira **Role of oxidative stress in the pathogenesis of nonalcoholic steatohepatitis** Free Radic Biol Med, 52 (2012), pp. 59-69

35. P. Letteron, B. Fromenty, B. Terris, C. Degott, D. Pessayre **Acute and chronic hepatic steatosis lead to in vivo lipid peroxidation in mice** J Hepatol, 24 (1996), pp. 200-208

36. Z. Yesilova, H. Yaman, C. Oktenli, A. Ozcan, A. Uygun, E. Cakir*, et al.* **Systemic markers of lipid peroxidation and antioxidants in patients with nonalcoholic Fatty liver disease** Am J Gastroenterol, 100 (2005), pp. 850-855

37. A. Ayala, M.F. Munoz, S. Arguelles **Lipid peroxidation: production, metabolism, and signaling mechanisms of malondialdehyde and 4-hydroxy-2-nonenal** Oxid Med Cell Longev, 2014 (2014), pp. 360438

38. E.B. Rankin, J. Rha, M.A. Selak, T.L. Unger, B. Keith, Q. Liu*, et al.* **Hypoxia-inducible factor 2 regulates hepatic lipid metabolism** Mol Cell Biol, 29 (2009), pp. 4527-4538

39. G.L. Semenza **HIF-1: mediator of physiological and pathophysiological responses to hypoxia** J Appl Physiol, 88 (2000), pp. 1474-1480

40. M.A. Cavadas, L.K. Nguyen, A. Cheong **Hypoxia-inducible factor (HIF) network: insights from mathematical models** Cell Commun Signal, 11 (2013), pp. 42

41. D.R. Van Harken, C.W. Dixon, M. Heimberg **Hepatic lipid metabolism in experimental diabetes. V. The effect of concentration of oleate on metabolism of triglycerides and on ketogenesis** J Biol Chem, 244 (1969), pp. 2278-2285

42. T. Matsumura, F.C. Kauffman, H. Meren, R.G. Thurman **O2 uptake in periportal and pericentral regions of liver lobule in perfused liver** Am J Physiol, 250 (1986), pp. G800-805

43. G.P. Mannaerts, L.J. Debeer, J. Thomas, P.J. De Schepper **Mitochondrial and peroxisomal fatty acid oxidation in liver homogenates and isolated hepatocytes from control and clofibrate-treated rats** J Biol Chem, 254 (1979), pp. 4585-4595

44. G. Finocchiaro, I. Colombo, S. Didonato **Purification, characterization and partial amino acid sequences of carnitine palmitoyl-transferase from human liver** FEBS Lett, 274 (1990), pp. 163-166

45. B. Gonzalez-Flecha, J.C. Cutrin, A. Boveris **Time course and mechanism of oxidative stress and tissue damage in rat liver subjected to in vivo ischemia-reperfusion** J Clin Invest, 91 (1993), pp. 456-464

46. N.J. Adimora, D.P. Jones, M.L. Kemp **A model of redox kinetics implicates the thiol proteome in cellular hydrogen peroxide responses** Antioxid Redox Signal, 13 (2010), pp. 731-743

47. A. Tovmasyan, C.G. Maia, T. Weitner, S. Carballal, R.S. Sampaio, D. Lieb*, et al.* **A comprehensive evaluation of catalase-like activity of different classes of redox-active therapeutics** Free Radic Biol Med, 86 (2015), pp. 308-321

48. L.D. Gauthier, J.L. Greenstein, B. O'rourke, R.L. Winslow **An integrated mitochondrial ROS production and scavenging model: implications for heart failure** Biophys J, 105 (2013), pp. 2832-2842

49. D. Del Rio, A.J. Stewart, N. Pellegrini **A review of recent studies on malondialdehyde as toxic molecule and biological marker of oxidative stress** Nutr Metab Cardiovasc Dis, 15 (2005), pp. 316-328

50. R.N. Hardwick, C.D. Fisher, M.J. Canet, A.D. Lake, N.J. Cherrington **Diversity in antioxidant response enzymes in progressive stages of human nonalcoholic fatty liver disease** Drug Metab Dispos, 38 (2010), pp. 2293-2301

51. F. Antunes, D. Han, E. Cadenas **Relative contributions of heart mitochondria glutathione peroxidase and catalase to H(2)O(2) detoxification in in vivo conditions** Free Radic Biol Med, 33 (2002), pp. 1260-1267
